# Temperature Dependence of Spin–Phonon Coupling in [VO(acac)_2_]: A Computational and Spectroscopic Study

**DOI:** 10.1021/acs.jpcc.1c06916

**Published:** 2021-09-30

**Authors:** Andrea Albino, Stefano Benci, Matteo Atzori, Laura Chelazzi, Samuele Ciattini, Andrea Taschin, Paolo Bartolini, Alessandro Lunghi, Roberto Righini, Renato Torre, Federico Totti, Roberta Sessoli

**Affiliations:** † Dipartimento di Chimica “Ugo Schiff” & INSTM RU, Universitá degli Studi di Firenze, Via della Lastruccia 3, Sesto Fiorentino, Florence 50019, Italy; ‡ European Laboratory for Non-Linear Spectroscopy (LENS), Universitá degli Studi di Firenze, Sesto Fiorentino, Florence 50019, Italy; § Laboratoire National des Champs Magnétiques Intenses (LNCMI), Univ. Grenoble Alpes, INSA Toulouse, Univ. Toulouse Paul Sabatier, EMFL, CNRS, F38043 Grenoble, France; ∥ Dipartimento di Chimica “Ugo Schiff” & Center of Crystallography, Universitá degli Studi di Firenze, Via della Lastruccia 3, Sesto Fiorentino, Florence 50019, Italy; ⊥ ENEA, Agenzia nazionale per le nuove tecnologie, l’energia e lo sviluppo economico sostenibile, Centro Ricerche Frascati, via Enrico Fermi 45, 00044 Frascati, Roma, Italy; # School of Physics, AMBER and CRANN Institute, Trinity College, Dublin 2, Ireland; ∇ Dipartimento di Fisica ed Astronomia, Universitá degli Studi di Firenze, Via G. Sansone 1, Sesto Fiorentino, Florence 50019, Italy

## Abstract

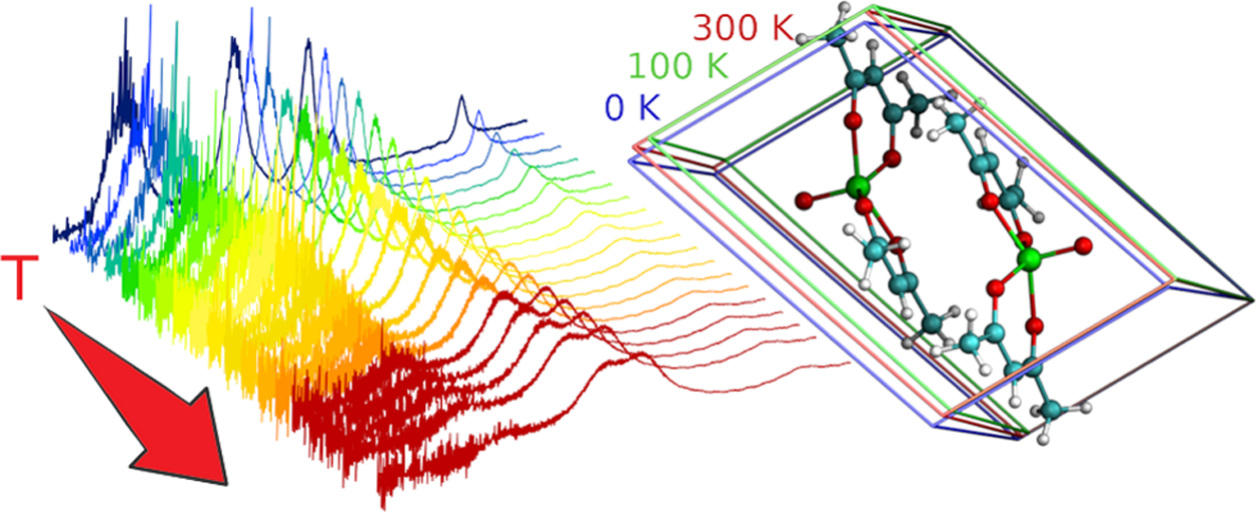

Molecular electronic spins are good candidates as qubits since they are characterized by a large tunability of their electronic and magnetic properties through a rational chemical design. Coordination compounds of light transition metals are promising systems for spin-based quantum information technologies, thanks to their long spin coherence times up to room temperature. Our work aims at presenting an in-depth study on how the spin–phonon coupling in vanadyl-acetylacetonate, [VO(acac)_2_], can change as a function of temperature using terahertz time-domain spectroscopy and density functional theory (DFT) calculations. Powder THz spectra were recorded between 10 and 300 K. The temperature dependence of vibrational frequencies was then accounted for in the periodic DFT calculations using unit-cell parameters measured at two different temperatures and the optimized ones, as usually reported in the literature. In this way, it was possible to calculate the observed THz anharmonic frequency shift with high accuracy. The overall differences in the spin–phonon coupling magnitudes as a function of temperature were also highlighted showing that the computed trends have to be ascribed to the anisotropic variation of cell parameters.

## Introduction

The quantum bit or qubit^1^ is the basic element of quantum information theory (QIT);^2^ it differs from its classical equivalent, the bit, since it can exploit the quantum superposition of the two states 0 and 1. Several systems are under investigation as potential qubits, e.g., electronic defects in diamond,^3^ photons,^4^ ion traps,^5^ superconducting circuits,^6^ and spins.^7−9^ Molecular electronic spin qubits are good candidates since they are characterized by a large tunability of their electronic and magnetic properties through a rational chemical design. Among them, coordination compounds of light transition metals, such as Ti(III), V(IV), and Cu(II),^10−12^ have demonstrated to be promising systems at least from the point of view of the superposition state’s lifetime. This quantity is determined by the spin–spin relaxation time, *T*_2_, but it is usually quantified through the phase memory time, *T*_m_, that is the measurable lower limit of *T*_2_.^13^ Another fundamental parameter in the evaluation of the qubit performance is the spin–lattice relaxation time, *T*_1_.^14^ If it is too short, it limits *T*_m_,^11^ preventing the implementation of more complex algorithms. In solid-state qubits, *T*_1_ is closely connected with lattice vibrations, i.e., phonons. Vibrations perturb spin degrees of freedom through the modulation of orbital contributions and the presence of spin–orbit coupling.

Therefore, the knowledge and characterization of phonons in solid-state qubit candidates are of crucial importance. A macroscopic crystal can present a huge number of atomic displacement degrees of freedom and, consequently, of vibrational modes. The use of space-group symmetry and the introduction of the reciprocal lattice allows a fundamental rationalization of this huge number of states. At the center of the Brillouin zone (BZ), the so-called Γ-point, i.e., for wavevector **k** = 0, the degrees of freedom are reduced to 3*N*, *N* being the number of atoms in the crystal unit cell so that the vibrational modes are 3*N* – 3 (3 are the rigid displacements of the unit cell, corresponding to zero frequency). Only the **k** = 0 vibrations can be measured with optical techniques at equilibrium; all of the remaining vibrational modes are classified, throughout the entire BZ, in 3*N* dispersion bands and are optically inaccessible. A peculiar feature of most molecular crystals is that intramolecular forces are much stronger than intermolecular ones so that the intramolecular vibrations in the crystal are almost unchanged from those of the isolated molecule. If the crystal unit cell contains *n* molecules, there are then 6*n* – 3 intermolecular phonons at **k** = 0, corresponding to rigid translations and rotations of the molecules. These “external” phonons have frequencies much lower (typically, less than 200 cm^–1^) than intramolecular vibrations. However, in some cases, where molecular vibrations of unusually low frequency are present, the separation of “internal” and “external” phonons is impossible and low-frequency phonon modes have mixed character.^15^ In the harmonic approximation, phonon modes are orthogonal to each other and their frequencies depend on temperature indirectly, as the thermal expansion of the lattice induces an elastic strain in the solid, which results in a shift (generally negative) of the phonon frequencies. The thermal expansion of crystals is a consequence of the anharmonicity of the crystal potential; however, it has been shown^16,17^ that the effect of thermal strain can be dealt with in a quasiharmonic approximation, adopting an effective crystal Hamiltonian that maintains the quadratic dependence on the atomic displacements. On the other hand, the anharmonic terms of the potential energy expansion enter directly into the description of the phonon dynamics as they mix different phonons and make phonon relaxation possible, thus determining the homogeneous linewidths of vibrational spectral lines. Furthermore, the same anharmonic terms cause additional temperature dependence of the frequencies through the temperature-dependent occupation number of phonons.

From a computational point of view, the harmonic treatment of molecular vibrations cannot explain all of the fine details of the vibrational spectra and the physical properties of these crystals that depend on the temperature.^18^ Density-functional perturbation theory or molecular dynamics simulations (from several ps to ns)^19^ are out-of-reach. The direct method of calculation of phonons^20^ here employed can be considered as a cheaper alternative. Unfortunately, even with this approach, the extension of lattice dynamics calculations beyond the second-order of expansion of the potential energy surface (PES) is severely limited by the enormous number of terms appearing in the expression of a large molecule; therefore, it is mandatory to assume some approximations when aiming to calculate higher orders of interaction.

In such a framework, an extensive characterization of the vibrational properties as a function of temperature becomes feasible and it is of paramount importance for the derivation of key chemical insights useful in the engineering of efficient molecular spin qubits. Indeed, as shown in other works,^10,21−27^ the study of phonons provides insights into the spin relaxation pathways pointing toward the rationalization of new synthetic strategies to achieve more performing systems.

The aim of this work is an in-depth study of the spin–phonon (hereafter, Sp-Ph for brevity) coupling in vanadyl-acetylacetonate, [VO(acac)_2_], both from a spectroscopic and theoretical point of view. Thanks to its simplicity (only 60 atoms in the unit cell), [VO(acac)_2_] has got the physique du rôle as a perfect training ground for extensive DFT simulations to investigate the rich vibrational properties of a molecular crystal.^26^ [VO(acac)_2_], already studied by inelastic neutron scattering,^28^ is here investigated by means of terahertz radiation in crystalline powder by using of an in-house built setup performing THz time-domain spectroscopy (THz-TDS).^29−31^

In our computational approach, the temperature dependence of the vibrational frequencies is accounted for using the unit-cell parameters measured at different temperatures. The results of this study confirm the paramount importance of low-energy phonons, where intramolecular and lattice characters are admixed.^15,25,26,32^

## Materials and Methods

### Crystal Structure

**Figure 1:**
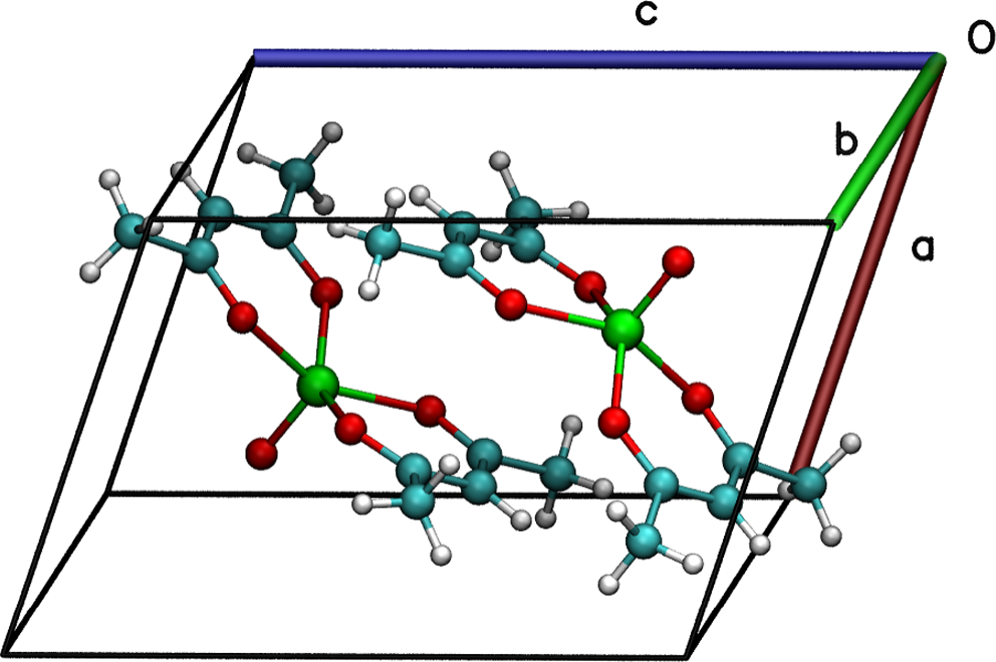
Primitive cell of [VO(acac)_2_] belonging to the triclinic *P*1̅ space group. Color code: vanadium, green; oxygen, red; carbon, cyan; and hydrogen, white.

The [VO(acac)_2_] metal center comprises a vanadyl ion VO^2+^ coordinated by two chelating bidentate β-diketonate ligands (Figure 1). The coordination geometry around the penta-coordinated V^IV^ metal center is a distorted square pyramid, as already reported in previous studies.^33^ This molecule crystalizes in a *P*1̅ space group (no. 2), for which the only point symmetry element is inversion. The asymmetric unit contains one molecule and the primitive unit cell contains two molecules, for a total of 60 atoms (see Table 1).

**Table 1:** Three Cell Parameter Sets Studied in This Work^a^

	***a***	***b***	***c***	α	β	γ	**vol**.
1_300K_	7.513	8.201	11.221	73.174	71.476	66.718	591.445
1_100K_	7.300	8.117	11.178	72.901	72.282	67.064	569.372
1_0K,cell–opt_	7.662	7.802	11.056	70.869	71.162	64.315	549.943

a Note that the cell parameters of 1_100K_ and 1_300K_ are the experimental ones while 1_0K,cell–opt_ are optimized by DFT. Lattice parameters are reported in Å and deg. The volume is in Å^3^.

The crystallographic structure has been already solved using high-resolution data in 1995 ^34^ at 294 K. Here, we present new data collected at 300 K, 1_300K_, and 100 K, 1_100K_, both on the same sample (Table S1). A good agreement between 300 and 294 K datasets is found, while the shrinking of cell parameters at 100 K is highlighted in Table 1.

### Experimental Apparatus and Data Analysis

THz spectra were measured by time-domain spectroscopy using a table-top experimental setup working in a transmission configuration. It allows one to achieve a very high signal-to-noise ratio, higher than what is usually obtained in the standard far-infrared investigation (FTIR). Furthermore, the nontrivial data analysis allows one to decouple the signals arising from the multiple reflections inside the sample, i.e., the Fabry–Pérot effect. The THz-TDS spectrometer is a home-built system based on optical laser pulses (T-light 780 nm fiber laser, MenloSystems) and low-temperature GaAs photoconductive antennas. The emitted THz radiation (pump) is collimated and focused on the sample by a couple of off-axis parabolic mirrors. The signal transmitted through the sample is again collimated and focused on the detector, a second photoconductive antenna, biased by a second optical pulse (probe). It results in a photocurrent that is measured by a lock-in amplifier and an acquisition board. The amplitude of the THz electric field is measured by varying the time delay between the pump and probe pulses so as to reconstruct its time profile. The homemade software in Matlab code was implemented to acquire the processed signal together with the reading of the delay line encoder and retraces the final time-dependent THz field. The whole THz setup is placed in a nitrogen-purged chamber to eliminate the strong absorption of water vapor. To improve the data quality, the effects of external perturbations during the acquisition (e.g., temperature fluctuations) measurements must be taken into account. For this purpose, measurements with and without a sample (reference) are cyclically repeated. We also implemented a new method of data analysis, described in detail in ref (29), that enables the extraction of the index of refraction, *n*, absorption coefficient, α, and thickness, *d*, of the material. This method is based on an iterative fitting process of the transmission parameters and it takes into account the Fabry–Pérot effect. More details on the description of the experimental setup and the data analysis procedure are reported elsewhere.^29^

### Sample Preparation

THz measurements were performed on two samples: (1_a_) a pellet of [VO(acac)_2_] and high-density polyethylene (HDPE) (1:1 mass ratio) prepared by milling vanadyl-acetylacetonate with HDPE powder (Merck CAS 42.901-5), the powder was pressed by means of a manual hydraulic press (about 0.8 GPa); (1_b_) a pure HDPE pellet on which we deposited a few drops of [VO(acac)_2_] dichloromethane solution of several microns by the drop-casting method.

### Computational Methods

#### Vibrational Properties

For the density functional theory-based calculations, we used the [VO(acac)_2_] crystallographic unit-cell coordinates. The temperature effects on the crystal structure were tested exploiting the X-ray data collected at 100 and 300 K. The availability of these data allowed to optimize the molecular structure keeping the experimental cell parameters fixed,^35^ in such a way the temperature effects can be indirectly accounted. The simulation of cell parameters at finite temperature can be accomplished only *via* dynamical DFT methods (*ab initio* molecular dynamics). Vibrational properties at 0 K were also computed by fully relaxing the electronic degrees of freedom, lattice constants, and atomic positions; the resulting crystal structure is hereafter referred to as 1_0K,cell–opt_. A tight DFT optimization is mandatory to avoid imaginary eigenvalues in the Hessian matrix. The different atoms in the unit cell are displaced by small amounts, and the forces on all other atoms are recorded to calculate the second derivative of the potential energy surface (PES) through numerical differentiation. Any atomic displacement **U**(**m**, μ) = ∑_*i*_*X*_*i*_(**m**, μ) – *X̅*_*i*_(**m**, μ), where *X̅*_*i*_(**m**, μ) is the equilibrium position, generates forces on all other atoms of the determined cell according to the relationship
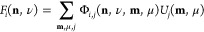
1where *n* and *m* are the primitive unit-cell indices, and *ν* and μ are the atomic indices. In the present study, *n* = 1 because only one primitive cell was included in the computation. This relates the forces generated to the force constant matrix Φ(**n**, ν, **m**, μ) and atomic displacement **U**(**m**, μ). We imposed a set of constraint equations on the force constant matrix, known as the acoustic sum rule, to ensure that the crystal energy is invariant under the global translation of the whole crystal (ω_acoustic_). The dynamical matrix is defined as^16,36^
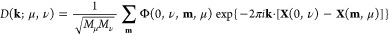
2Here, *M*_μ_ and *M*_ν_ are masses of atoms, and **k** is the wave vector. The solution of the eigenproblem (eq 3) gives eigenvalues ω^2^(**k**, *j*) and eigenvectors **e**(**k**, *j*) of 3*N* orthogonal normal modes of vibrations
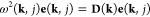
3The calculation is restricted to one primitive cell, giving access only to 3*N* – 3 zone-center phonons (at the Γ-point).^37^

The Γ-point approximation comes with no loss of information. The full inclusion of the Brillouin zone integration, important to quantitatively estimate the optical phonon bands, is not part of the main focus of the present work.^25^

A more than fair approximation in the analysis of phonons’ decay mechanisms is achieved limiting the calculation to the DFT Γ-point optical modes, along with the acoustic phonons in the whole Brillouin zone. The latter were already computed by some of us at the force field (FF) level,^33^ at the DFT level with the finite displacement method at Γ-point,^25^ and in the Brillouin zone.^28^

Periodic *ab initio* calculations of energy and forces were performed using the Quickstep module^38^ present in the CP2K atomistic simulation package.^39^

GTH pseudopotentials^40−42^ for the core electrons and valence pseudo-wavefunctions are expanded in Gaussian-type orbitals, and the density is represented in a plane wave auxiliary basis set. In particular, for V, O, C, and H elements, the DZVP-MOLOPT-SR-GTH basis set was used.^38^

The Perdew–Burke–Ernzerhof (PBE) parametrization^43^ was chosen for the calculation. A pairwise dispersion correction proposed by Grimme^44−46^ was included in DFT calculations. The Broyden–Fletcher–Goldfarb–Shanno (BFGS) minimizer^47,48^ was employed to carry out the structure optimization. The convergence of the plane wave basis cut-off was reached for 10 000 *Ry* with a convergence threshold of 1.0 × 10^–9^ au for the SCF energy and 5.0 × 10^–8^ au/Å for the forces. A cut-off of 10 000 *Ry* is cumbersome and the best compromise between accuracy and cpu-time consumption was chosen to be 5000 *Ry* (see Figures S1 and S2). Such a choice was taken also on the basis of the convergence of vibrational energies in the low-energy range (0–100 cm^–1^). Indeed, the error between the frequency values computed for 10 000 and 5000 *Ry* is just a few decimal digits of the wavenumber. The high level of accuracy chosen precluded to calculate the phonons’ dispersions at the DFT level for all of the three structures investigated.

#### Magnetic Properties and the Spin–Phonon Coupling

The method to investigate the Sp-Ph coupling has been developed in previous works^15^ and is based on a perturbative formalism. Calculation of the Sp-Ph Hamiltonian parameters was carried out with the ORCA package.^49^ The level of theory chosen is DFT and the PBE0 hybrid functional^50^ was used throughout the computation. A balanced polarized triple-ζ basis set was adopted (def2-TZVP) for V and S atoms, while a polarized single-ζ basis set (def2-SVP) was used for light elements such as C and H. The geometrical equivalence between the two molecules present in the crystal cell after p-DFT optimization with CP2K was ascertained. An RMSD of 0.12 was computed on V, O, and C atoms (0.94 on V, O, C, and H atoms) indicating that two molecules can be considered equivalent even after a symmetry-unconstrained optimization. For such a reason, only one molecule was used for the Sp-Ph calculations. The unperturbed Hamiltonian is evaluated with respect to external perturbation, sampling six points along each coordinate (see Figure S3). The *g* vs *q*_α_ plot is then fitted with a polynomial whose first-order coefficient directly yields the first-order derivative .

The  for a *S* = 1/2 spin system, neglecting the hyperfine term that plays a key role only at the low magnetic field,^11^ contains only the Zeeman term , where the main parameter depending on ionic coordinates is the second-order correction to the Landé factor *g*, μ_b_ is the Bohr magneton, and *B* is the magnetic field.

The Sp-Ph coupling Hamiltonian connects molecular vibrations to the spin via the spin–orbit interaction,^51,52^ and, in the weak coupling assumption, it is linear in the ionic displacements  and it reads
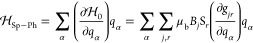
4The dynamics of the whole system (spin and phonons) can be studied by the Liouville–von Neumann equation for the density operator, within the approximations described by Redfield^53^ in the framework of the open quantum system theory.^54^ The spin–phonon coupling coefficients constitute the main ingredients to describe the interplay of the principal system (spin degrees of freedom) and the ancillary system (surrounding environment of the phonons), as shown in previous works.^26^

## Results and Discussions

### THz Spectra of Powder Samples

(1_a_) and (1_b_) have been prepared according to two different procedures to check that the applied pressure, essential to make the pellet, does not induce any phase transition or changes in the crystal structure. The THz spectra of (1_a_) and (1_b_) are shown in Figure S4.

The two spectra are identical, except for the intensity that is related to the different concentrations of vanadyl-acetylacetonate in the samples. No evident effects of the applied pressure occur; the same result is observed by PXRD investigation on (1_b_) compared with the vanadyl-acetylacetonate powder and pure HDPE, as shown in Figure S4. Therefore, hereafter, the discussion will be focused on (1_a_).

**Figure 2:**
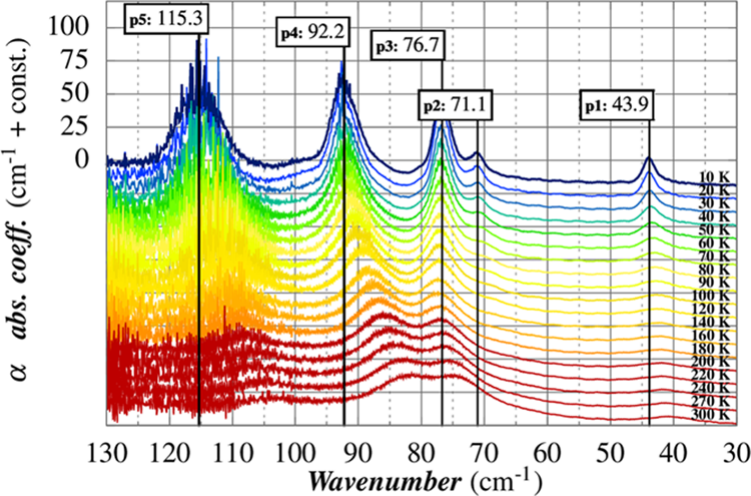
THz spectra of (1_a_) as a function of temperature.

Figure 2 shows the THz spectra measured in the temperature range 10–300 K. At the lowest temperature, five peaks (p1–5) are detected. As the sample temperature is increased, all of them become broader and most of them shift to lower energy. It is worth noting that the lowest phonon detected falls at 43.9 cm^–1^ (p1), which is exactly the value predicted by the Brons–van Vleck model applied to the spin relaxation rate in our previous work.^21^

**Figure 3:**
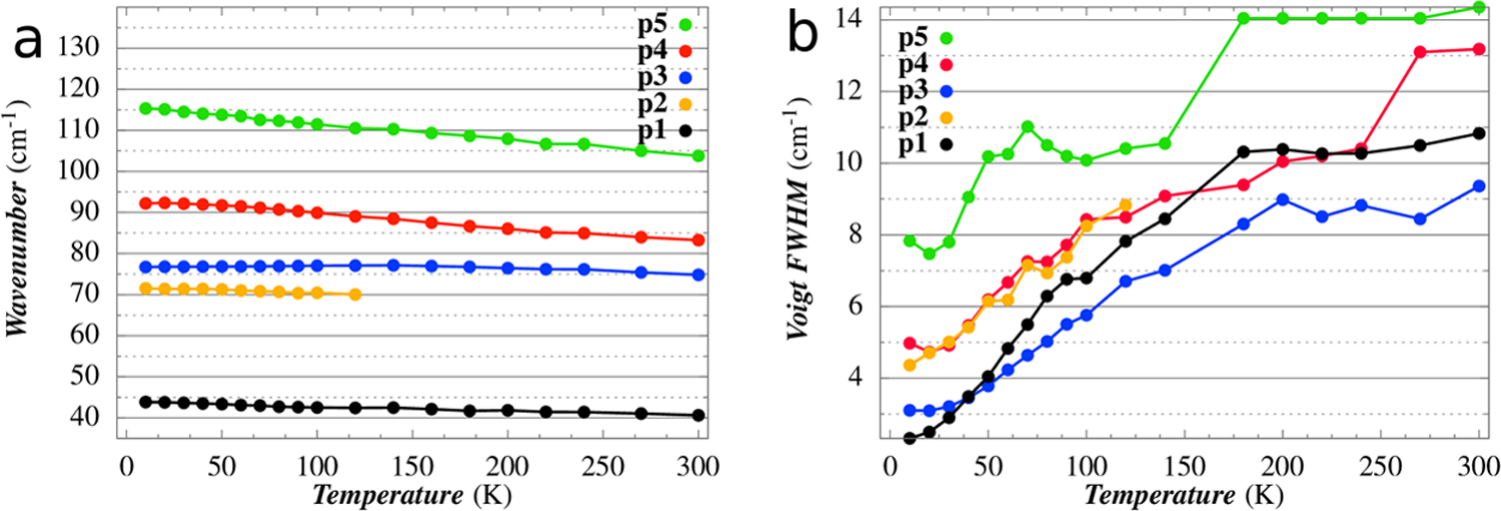
(Left) Temperature dependence of the peaks’ frequency. (Right) Temperature dependence of linewidths obtained using a Voigt fit function for each peak and a baseline correction.

The extraction of peaks’ parameters, i.e., central frequency and linewidth (Γ_lw_), has been performed by fitting each of them with a pseudo-Voigt function. Moreover, the baseline correction has been taken into account. Figure 3 summarizes the temperature dependence of the five bands. The error analysis on the five peaks’ position led to an average standard deviation of less than 1%. The weak peak at ca. 71 cm^–1^ (p2) is not detectable above 120 K, as it becomes too broad and the strong peaks at higher frequency get closer and broader. The general trend is a decrease of frequency when the temperature increases and it is ascribed primarily to the effect of the thermal expansion of the lattice, which results in the softening of the intermolecular forces.^16^

The peak at ca. 77 cm^–1^ (p3) is the only one that deviates from the general trend: its temperature dependence is very weak and not monotonous. The nature of the computed mode which should correspond to p3 (*vide infra*) is characterized by an in-phase rigid vibration of the external acac ligands of the two molecules in the cell with the ones belonging to the molecules in the neighbor cell replica (see Figure S7). Therefore, the variation of cell parameters as a function of temperature is expected to have a very limited impact on this mode.

Figure 3 shows how the linewidth of each peak, in terms of pseudo-Voigt, varies by increasing the temperature from 10 to 300 K. A deconvolution process was carried out to separate the Lorentzian contribution (Γ_L_) and the Gaussian one (Γ_G_). The first contribution takes into account the homogeneous broadening, connected to the phonon–phonon and phonon–lattice interactions, through the anharmonic terms of the crystal potential expansion, whereas the second one takes into account the inhomogeneous broadening, which is predominantly attributed to the variation of frequency with the phonons wave vector (**k**) direction of the phonons active in IR. Indeed, in common spectroscopic techniques, e.g., IR and Raman, the conservation of the moment is fulfilled in a small surrounding of the Γ-point (**k** ≃ 0), making the phonons’ frequency dependent on the **k** direction.

**Figure 4:**
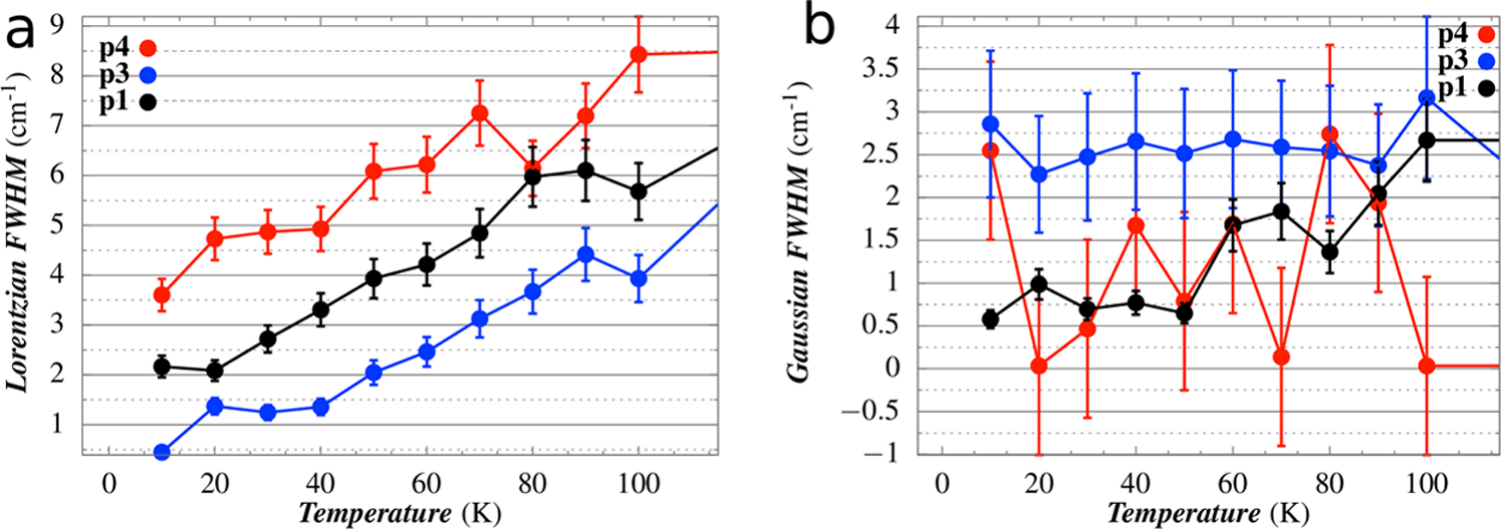
Temperature dependence of (a) Lorentzian component and (b) the Gaussian component of the Voigt profile of each peak.

The results collected in Figure 4 show that, for the temperature range 10–100 K, the homogeneous broadening of peaks p1, p3, and p4 increases almost linearly with temperature, while the inhomogeneous width is practically constant. The relative standard deviation is of the order of 10–20% since Γ_G_ and Γ_L_ are particularly sensitive to the irregularity of the peak tails. In addition, the loss of a well-defined shape of p2 above 70 K and the high noise that affects p5 do not allow to deduce a clear temperature trend for these peaks.

### Calculated Vibrational Spectra

In the Introduction section, we pointed out that the availability of X-ray data collected at cryogenic and room temperature (100 and 300 K) allowed us to fix the experimental cell parameters in the DFT energy minimization, prompting the estimation of the errors introduced in the vibrational analysis when the temperature effects on the structure are neglected, i.e., when cell parameters are free parameters in the energy minimization procedure. We denote 1_0K,cell–opt_, 1_100K,opt_, and 1_300K,opt_ as the DFT optimized structures; in 1_0K,cell–opt_, both cell parameters and atomic coordinates are optimized while, in 1_100K,opt_ and 1_300K,opt_, only atomic coordinates were left to relax keeping the cell parameters fixed to experimental values. Lowering the temperature from 300 to 0 K, a contraction occurred along the *b* and *c* axes, while a nonmonotonous expansion along the *a-*axis was observed. The overall variation of the cell parameters led to a unit-cell volume contraction. 1_0K,cell–opt_ was found to be more stable than 1_100K,opt_ of 2.80 kcal/mol, while 1_100K,opt_ was more stable than 1_300K,opt_ of 0.92 kcal/mol, as expected for the most enthalpic favored structure. The full relaxation of 1_0K,cell–opt_ led to optimized cell parameters that show a maximum deviation with respect to 1_100K_ of 4.8 and 2.8% for lengths and angles of lattice constants, respectively. The volume underwent a shrinking of ca. 5%. Even more, it is worth highlighting that *a*, β, and γ parameters computed for 1_0K,cell–opt_ do not completely follow the expected trend indicated by the experimental parameters obtained for 1_100K_ and 1_300K_ unit cells. These results have also a general relevance, as they show that neglecting the temperature effects on cells, i.e., using static DFT cell parameters optimized at 0 K, a poorer agreement in reproducing the vibrational spectra at finite temperatures can be attained. See, for instance, the computed p1_C_, which is shifted to almost 20 cm^–1^ from 0 to 300 K calculated phonons.

**Figure 5:**
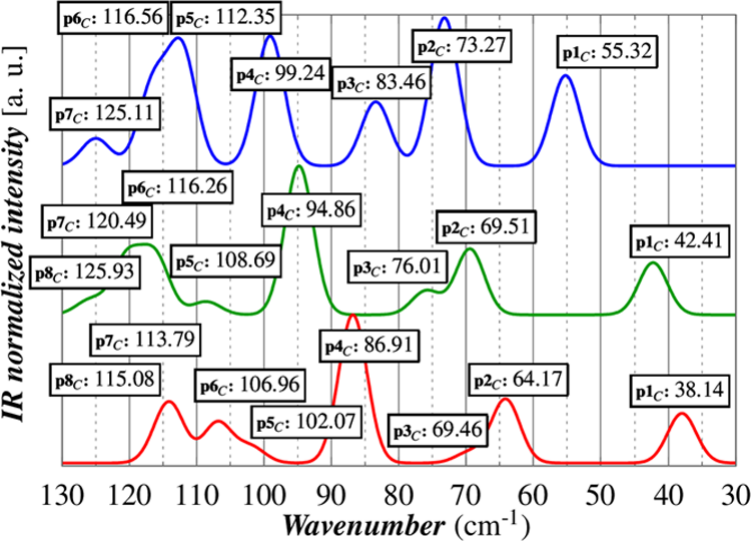
DFT vibrational analysis of 1_0K,cell–opt_ (blue), 1_100K,opt_ (green), and 1_300K,opt_ (red). A direct comparison of experimental and calculated spectra is available in the SI (Figure S5).

**Figure 6:**
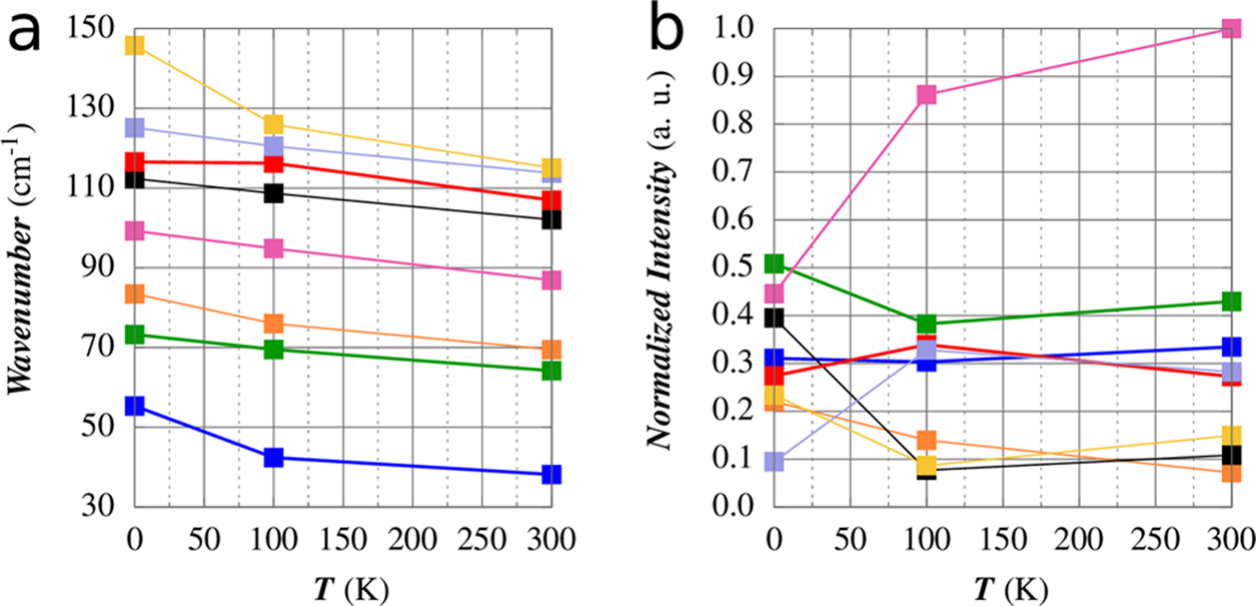
Calculated frequencies (a) and intensities (b) of the lowest 8 IR-active modes.

The computed IR spectra for the three cells are reported in Figure 5. The frequencies of the lowest 20 Raman-active (9) and IR-active (11) modes are reported in Table 2 with the trends for the first IR modes gathered in Figure 6. The full spectral data are reported in Table S3.

**Table 2:** Frequencies of the Lowest 20 Raman- and IR-Active Modes^a^

IR normal modes											
1_0K,cell–opt_				**1**_**100K,opt**_				**1**_**300K,opt**_			
α	ν_α_ (cm^–1^)	int. (au)	Sp-Ph (au)	α	ν_α_ (cm^–1^)	int. (au)	Sp-Ph (au)	α	ν_α_ (cm^–1^)	int. (au)	Sp-Ph (au)
6	55.323	0.311	2.331 × 10^–7^	4	42.411	0.303	3.905 × 10^–7^	4	38.137	0.335	3.787 × 10^–7^
8	73.267	0.509	4.399 × 10^–7^	9	69.518	0.383	8.549 × 10^–7^	9	64.171	0.430	8.199 × 10^–7^
11	83.461	0.220	2.453 × 10^–7^	10	76.013	0.140	4.069 × 10^–7^	10	69.467	0.072	2.448 × 10^–7^
13	99.240	0.446	3.330 × 10^–7^	12	94.864	0.862	6.695 × 10^–7^	12	86.907	1.0	6.289 × 10^–7^
15	112.346	0.395	5.968 × 10^–7^	15	108.685	0.0769	3.620 × 10^–7^	15	102.072	0.109	2.255 × 10^–7^
17	116.558	0.274	2.171 × 10^–7^	17	116.262	0.339	4.334 × 10^–7^	17	106.960	0.272	4.870 × 10^–7^
18	125.108	0.0947	2.842 × 10^–7^	18	120.492	0.328	1.231 × 10^–7^	18	113.787	0.283	6.313 × 10^–8^
20	145.745	0.234	8.805 × 10^–8^	20	125.933	0.087	2.795 × 10^–7^	19	115.077	0.149	4.863 × 10^–7^

Raman normal modes											
1_0K,cell–opt_				**1**_**100K,opt**_				**1**_**300K,opt**_			
α	ν_α_ (cm^–1^)		Sp-Ph (au)	α	ν_α_ (cm^–1^)		Sp-Ph (au)	α	ν_α_ (cm^–1^)		Sp-Ph (au)
4	43.313		1.130 × 10^–7^	5	46.320		1.430 × 10^–7^	5	40.497		2.427 × 10^–8^
5	50.872		1.648 × 10^–7^	6	48.492		3.037 × 10^–7^	6	43.222		3.545 × 10^–7^
7	63.349		6.260 × 10^–7^	7	57.788		7.608 × 10^–7^	7	53.353		5.580 × 10^–7^
9	75.290		2.357 × 10^–7^	8	66.991		3.717 × 10^–7^	8	61.592		3.906 × 10^–7^
10	81.218		3.927 × 10^–7^	11	79.998		9.584 × 10^–8^	11	73.577		1.711 × 10^–7^
12	98.285		4.581 × 10^–7^	13	100.610		9.038 × 10^–7^	13	89.691		6.472 × 10^–7^
14	111.392		2.764 × 10^–7^	14	104.128		4.613 × 10^–7^	14	96.560		4.950 × 10^–7^
16	114.618		2.050 × 10^–7^	16	110.094		1.703 × 10^–7^	16	102.096		3.003 × 10^–7^
19	135.847		2.568 × 10^–7^	19	124.879		8.979 × 10^–8^	20	115.669		6.292 × 10^–8^

a α is the normal mode index, ν_α_ is the frequency, int. is the IR intensity, and Sp-Ph is the magnitude of the spin–phonon coupling coefficient.

The computed redshifts of peaks in the range 30–130 cm^–1^ are in an overall good agreement with those observed for the THz spectra measured at 10, 100, and 300 K. A one-to-one correspondence with the experimental peaks is valid for p1–4; the comparison is impossible for p5, as this band results, according to our calculation, as the superposition of four peaks (p5_C_–8_C_). The calculated relative intensities are also in good agreement with the experimental ones with the only exception for p2 and p3, for which an inversion of their intensities can be claimed. This interpretation is more plausible than an energy swap of the two modes because p3_C_ shows a larger shift as a function of the temperature than p2_C_ as, indeed, experimentally observed. The comparison of the calculated linewidths at different temperatures is impracticable, as in our approach the effects of temperature are taken into account only using different cell parameters.

**Figure 7:**
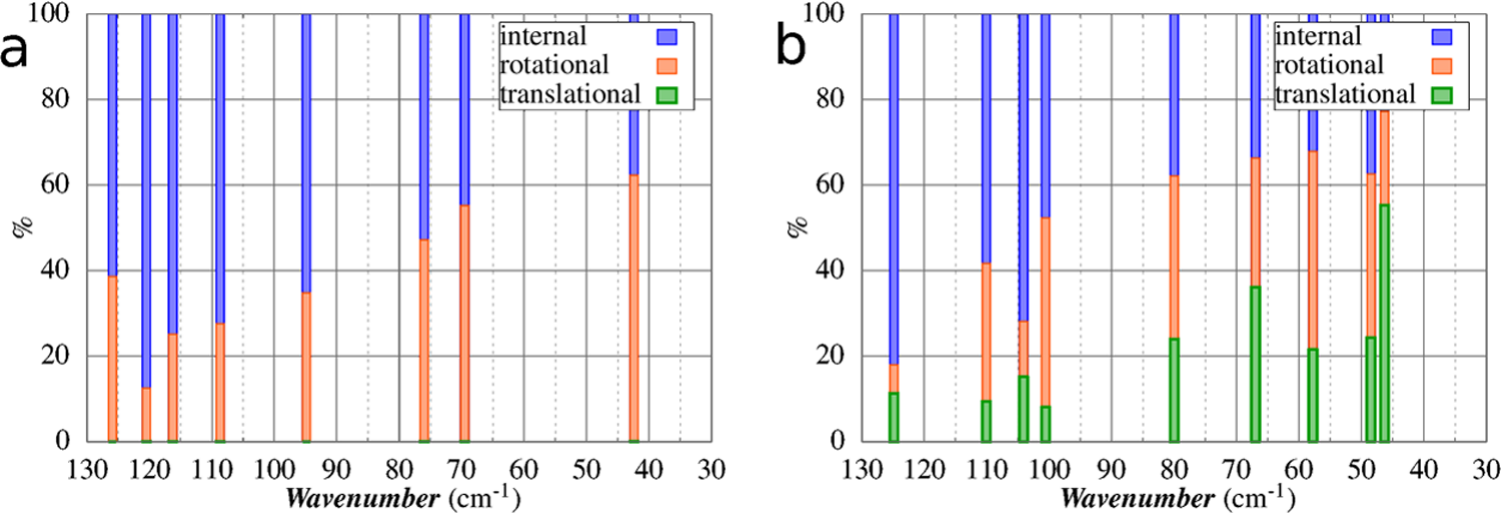
Total molecular motion associated with (a) IR and (b) Raman normal modes from the 1_100K,opt_ set in the low wavenumbers range (30–130 cm^–1^) decomposed in intramolecular percentage (blue), rotational percentage (orange), and translational percentage (green). In Figure S10, we show the plots of the first vibrational modes composition for each temperature.

In Figure 7, we report the decomposition procedure outlined by Neto et al.^55^ and developed by Lunghi et al.^15^ to compute the amplitude of local translation, local rotation, and internal vibrations of a single [VO(acac)_2_] molecule inside its crystal. The nature of the lowest eight peaks shows that the reticular contributions decrease as the energy of modes increases, as expected. Such a contribution is still detectable up to the 20th mode with a 20–30% of external character. The modes can be sketched as the following:

(1) in p1_C_, two [VO(acac)_2_] molecules rigidly tilt one with respect to the other (Figure S6).
(2) p2_C_–3_C_ can be considered quasidegenerate modes where the two acac ligands of a [VO(acac)_2_] molecule tilt out-of-phase (p2_C_) or in-phase (p3_C_) with respect to one acac belonging to a neighboring [VO(acac)_2_] molecule: in p2_C_, the neighboring molecule is inside the unit cell; in p3_C_, in an adjacent one (Figures S6 and S7).
(3) in p4_C_–8_**C**_, the vanadyl group bending and acac oxygen wagging contributions became significant (Figures S7, S8, and S9)

#### Phonon Decay Mechanisms from Linewidth Analysis

The analysis of the temperature dependence of the homogeneous linewidth allows the rationalization of the decay mechanism of the phonons and the estimation of the third-order anharmonic coefficients.

The temperature-dependent anharmonic coupling of phonons leads to relaxation via multiphonon processes.^56^ In the simplest case (predominant at low temperature), line broadening results from two-photon decay, involving down- and up-conversion mechanisms. In the first case, the optical phonon decays into two phonons of lower frequencies (down-conversion); in the second case, the optical phonon and a phonon of the thermal bath add up to produce a more energetic phonon (up-conversion).^56^ Energy and momentum conservation are required for all processes. In the limit *kT* > *hν* (where *ν* is the frequency of the optical phonon), a linear trend of the Lorentzian linewidth indicates that two-phonon decay processes are the dominant relaxation mechanism.

The decay pathway identified for p1, p3, and p4 corresponds to the down- and up-conversion processes. The simplest down-conversion process fulfilling the conservation of energy and momenta requires that the energy of generated phonons is half of the starting one, 2*h*ν = *h*ν_0_. If we assume, for instance, that the optical phonon ν_0_ decays into two phonons of the same frequency *ν*, and the phonons ν_1_ and ν_2_ sum up to create a ν_0_ optical phonon, we can write the following equation^56,57^
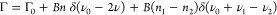
5where Γ_0_ is the residual linewidth at zero temperature, *n* = [exp(*h*ν/*kT*) – 1]^−1^ is the occupation number of the phonon involved in the decay at temperature *T*, and *B* is the third-order anharmonic coefficient; the Kronecker δ functions ensure energy conservation.

In principle, the anharmonic lattice dynamics theory provides the recipes for the calculation of phonon linewidth, which requires knowledge of the phonon frequencies in the entire Brillouin zone. This goal goes far beyond the limits of this work; we adopt here a very simplified approach that involves drastic approximations. We verified that the up-conversion mechanism gives a small contribution for p1_C_, while larger contributions are found for p3_C_ and p4_C_. In addition, we assume that the down-conversion mechanism in which two phonons of the same frequencies are created can be taken as representative of all the down-conversion processes.

**Figure 8:**
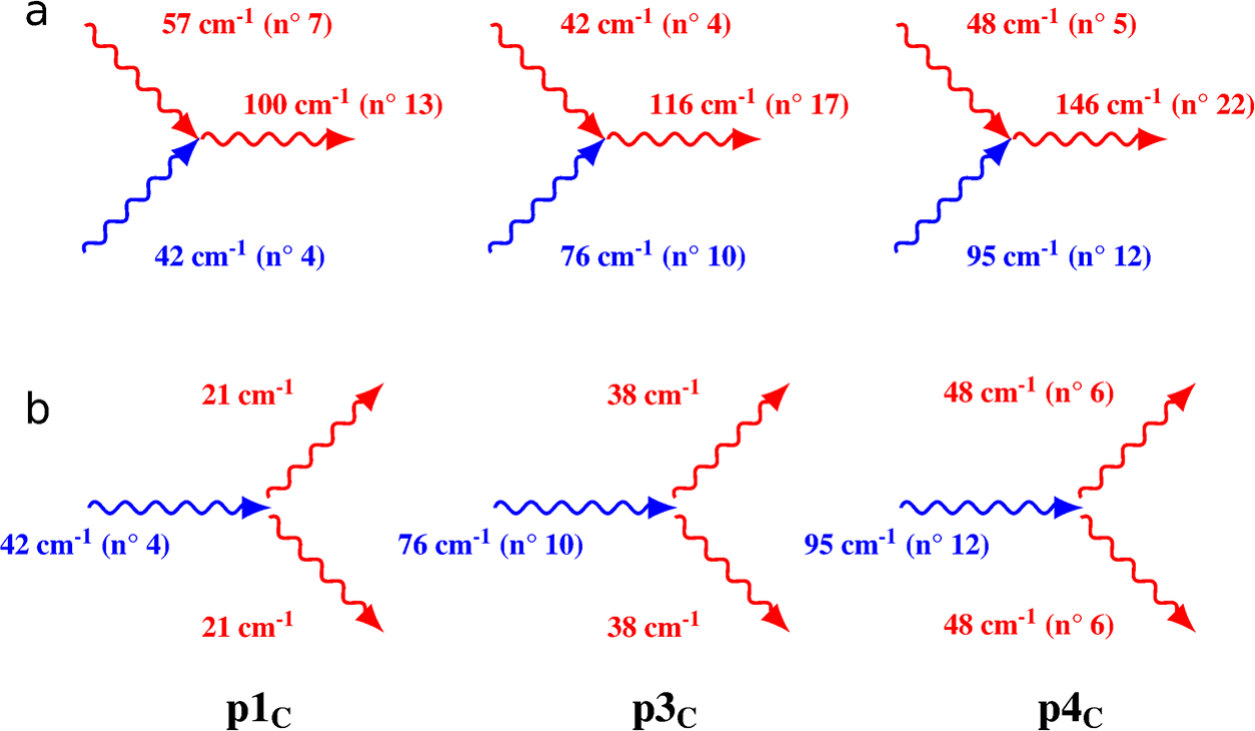
Two-phonon up- (a) and down-conversion (b) processes. The optical phonons’ frequencies are chosen among the 1_100K,opt_ set. The blue phonons refer to the ones appearing in the THz spectrum and the red ones are those IR or Raman modes participating in the multiphonon relaxation processes.

Within these limits, for p1_C_ up-conversion, might be allowed considering the interaction with the Raman-active mode 7 (57 cm^–1^) to create a Raman-active phonon at 100 cm^–1^ (mode 13). p3_C_ interacts with p1_C_ and creates a phonon p6_C_ at 116 cm^–1^. The matching is fulfilled considering that the tail of p6_C_ extends up to 125 cm^–1^. Phonon p4_C_ can combine with Raman mode 5 (48 cm^–1^) to generate a phonon at 146 cm^–1^. Concerning the down-conversion processes, the two lowest optical phonons p1_C_ and p3_C_ can decay only into acoustic phonons, which, as previously reported,^28^ extend from 0 to ca. 40 cm^–1^ at the Brillouin zone boundary. The relaxation of p4_C_ involves two phonons of 48 cm^–1^, a frequency value at the lowest limit of the optical phonon branches. This schematic description of the relaxation processes is summarized in Figure 8.

The fitting to the experimental linewidth in the temperature range 10–100 K gives for p1_C_ a third-order anharmonic coefficient *B* of ca. 2 cm^–1^, with a negligible contribution from the up-conversion term. Conversely, the *B* value for p3_C_ and p4_C_ is ca. 2 cm^–1^ (2.5 and 4 cm^–1^, respectively, considering only the down-conversion process).

### Spin–Phonon Coupling Analysis

**Figure 9:**
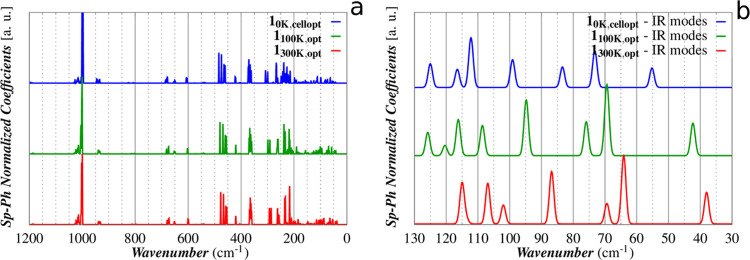
Comparison between coupled modes at different temperatures: (a) the DFT Sp-Ph coupling interaction in the 0–1200 cm^–1^ spectral range; (b) zoom on the 30–130 cm^–1^ spectral range.

A first qualitative fingerprint of interaction between magnetism and vibrations in each molecule requires the calculation of the phonon spectrum and the amplitude of Sp-Ph coupling for all possible modes, , as depicted in the previous section. The computed Sp-Ph couplings for all of the energy range (0–1200 cm^–1^) for both Raman and IR modes are reported in Figure 9 for all of the three considered cells. Unfortunately, the lack of the inversion center in the molecule did not allow to fully exploit symmetry considerations in the rationalization of the normal modes, as evidenced in previous works.^32^

As stated in the previous section, some cell parameters computed for 1_0K,cell–opt_ do not completely follow the expected trend indicated by the experimental parameters obtained for 1_100K_ and 1_300K_ unit cells. The different cell parameters led to an alteration of the compositions of the normal modes and, therefore, to their Sp-Ph couplings. A closer similarity of 1_100K_ vs 1_300K_ Sp-Ph coefficients is indeed found, in contrast to 1_100K_ vs 1_0K,cell–opt_ and 1_300K_ vs 1_0K,cell–opt_.

The spectrum can be divided into two regions. Above 300 cm^–1^, the coupled modes are dispersedly grouped for all frequency ranges with very similar distributions among the three different cells. The group of vibrations located at 1000 cm^–1^ showed the strongest Sp-Ph coefficients. Indeed, the involved normal modes are characterized by a relevant contribution of the characteristic V = O_vanadyl_ stretching. Strong couplings are also observed for groups centered at 470 cm^–1^ (O_acac_–V–O_acac_ symmetric stretching) and 365 cm^–1^ (O_acac_–V–O_acac_ symmetric stretching). The temperature effects on the Sp-Ph coupling magnitudes are not evident in this frequency range. Below 300 cm^–1^, a denser distribution of coupled modes was computed. The most coupled modes are in the 200–300 cm^–1^ energy window, and a variation of the magnitude as a function of temperature is now observed. The largest dependence (a factor 5 or higher between 0 and 300 K) was computed for the Raman mode 5 (46.32 cm^–1^ at 100 K, symmetric translation of molecules) and IR modes 18 (120.49 cm^–1^ at 100 K, V–O_acac_ bendings), 45 (260.53 cm^–1^ at 100 K, O_acac_–V = O_vanadyl_ wagging and twisting), and 47 (264.08 cm^–1^ at 100 K, O_acac_–V–O_acac_ wagging). For the first three modes, by increasing the temperature, a decrease of the Sp-Ph magnitude was observed. An opposite behavior was observed for the fourth, instead. For several other modes, a decrease or increase in their Sp-Ph coupling was observed though, even if the variation in temperature was not so pronounced. These differences can be ascribed to the modified perturbation of the first coordination sphere caused by the variation of the primitive cell parameters observed for the three considered temperatures (see Table 1). The nonmonotonous (a, β, γ) and monotonous (*b*, *c*, α) trends of the cell parameters can differently alter the composition of the normal modes and modify their Sp-Ph coupling efficiency.

Focusing our attention on the IR modes in the experimental THz range (30–130 cm^–1^), the differences between the computed Sp-Ph dispersions of 1_0K,cell–opt_ and 1_100K_/1_300K_ are more evident.

The computed Sp-Ph coefficients are reported in Figure 9, and only 1_100K,opt_ and 1_300K,opt_ will be discussed for homogeneity.

The Sp-Ph coupling can decrease its magnitude or remain unaffected with the increase of temperature; its variation is not constant for each peak going from a slight variation of the Sp-Ph coupling for p1_C_ (rigid tilting of molecules) to a significant one for p3_C_ and p5_C_ (first coordination sphere distortion).

**Figure 10:**
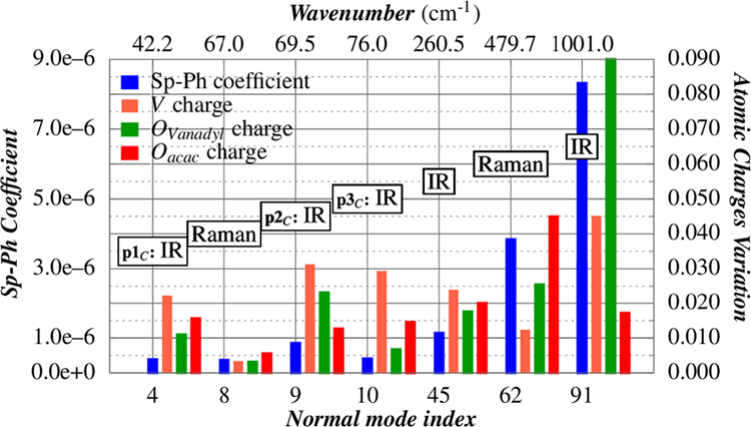
Charge variation of one molecule of [VO(acac)_2_] crystal is analyzed along several normal modes from the 1_100K,opt_ structure. The metal center and first coordination sphere atomic charges are reported. The data are reported in Table S5, while the phonons’ composition is depicted in Figure S10.

In a recent paper, some of us have shown for the [Dy(acac)_3_(H_2_O)_2_]^58^ complex that a large Sp-Ph coupling is found for those normal modes that can induce large polarization on the atoms of the first coordination sphere. Even in the case of [VO(acac)_2_], such a statement is confirmed. In detail, the normal modes 45, 62, and 91 (depicted in Figure S11), which involve the largest charges variation, show the largest Sp-Ph coupling (see Figure 10).

The indications from the computed results are twofold. From a strictly computational point of view, our analysis shows that the use of static approaches for the description of the phonon distribution should be performed when the temperature effects can be indirectly accounted for the use of crystallographic cells collected at different temperatures. We have shown that in such a framework, a quantitative agreement with the THz experiment was obtained, while the ordinary approach of optimizing the geometries along with or without the crystallographic cell parameters can lead only to qualitative results for the vibrational spectrum and for the Sp-Ph coupling. From a structural point of view, it is possible to explicitly show how the Sp-Ph coupling associated with a single normal mode can vary as a function of temperature as a direct consequence of the variation with the temperature of the crystal cell parameters. It is also evident that the Sp-Ph relaxation mechanism can give rise to a more complex behavior than the polynomial or exponential functions usually considered in the literature^59,60^ for the *T*_1_ temperature dependence.

## Conclusions

The harmonic approximation of molecular vibrations is highly valuable for interpretation of the main features of the vibrational spectra but it cannot explain all their fine structures, which depend on the temperature. At finite temperatures, anharmonic motions of the molecules must be considered since they allow for the possibility of interactions among the harmonic phonons, resulting in slight changes in the energy and composition of characteristic vibrations. The most straightforward parameters to be considered as modified by anharmonicity are the cell parameters. The crystal structures obtained from X-ray diffraction collected at cryogenic and room temperatures allowed us to calculate the observed THz anharmonic frequency shift with high accuracy.

The overall differences in the spin–coupling magnitudes as a function of temperature were highlighted. It turned out that Sp-Ph coupling can decrease or increase its magnitude with the increase of temperature. The reason for such trends has to be ascribed to variations of primitive vectors and unit-cell angles observed for the two considered temperatures. Indeed, the cell volume tunes the normal modes modifying the perturbation to the first coordination sphere of the vanadium ion. The overall results presented in this paper have also a general relevance allowing the quantification of the errors introduced by neglecting the temperature effects on the cell parameters. The cell parameters obtained by full optimization at 0 K do not follow the trends outlined by 100 and 300 K experimental structures, suggesting the possibility that a constrained optimization employing the experimental cells could yield more accurate results.

Moreover, the presented results can have a general relevance regarding the calculation of the *T*_1_ values, too. Indeed, even if significant results have been recently achieved in its computation at the ab initio level,^25,26^ the *T*_1_ temperature dependence was only due to the phonon thermal distribution, keeping the Sp-Ph coupling as temperature-independent. However, our calculations suggest that even though the computed Sp-Ph coupling variations are small, the overall calculated variation becomes mandatory for a more rigorous reproduction of *T*_1_ along a wide temperature range.

Therefore, the results of this study represent further pieces of the puzzle of the role played by vibrations in determining the relaxation properties of potential molecular qubits at the quantitative level. In particular, the deeper knowledge of the Sp-Ph coupling might allow one to exploit phonons for the accurate control, e.g., initialization and readout of the qubits. This can be performed by using THz pulses by means of time-resolved ultrafast spectroscopy.

## Supporting Information Available

X-ray crystallographic data; convergence test; cartesian derivatives comparison; full spectral data; normal-mode composition; atomic charges (PDF)

## References

[ref1] Nielsen M. A.; Chuang I. L. Quantum Computation and Quantum Information; Cambridge University Press: Cambridge, UK, 2011.

[ref2] Grazioso F. Introduction to Quantum Information Theory and Outline of Two Applications to Physics: The Black Hole Information Paradox and the Renormalization Group Information Flow. arXiv preprint arXiv:1507.00957v2, 2015.

[ref3] Balasubramanian G.; Neumann P.; Twitchen D.; Markham M.; Kolesov R.; Mizuochi N.; Isoya J.; Achard J.; Beck J.; Tissler J.; et al. Ultralong spin coherence time in isotopically engineered diamond. Nat. Mater. 2009, 8, 383–387. 10.1038/nmat2420. 19349970

[ref4] Knill E.; Laflamme R.; Milburn G. J. A scheme for efficient quantum computation with linear optics. Nature 2001, 409, 46–52. 10.1038/35051009. 11343107

[ref5] Blatt R.; Wineland D. Entangled states of trapped atomic ions. Nature 2008, 453, 1008–1015. 10.1038/nature07125. 18563151

[ref6] Clarke J.; Wilhelm F. K. Superconducting quantum bits. Nature 2008, 453, 1031–1042. 10.1038/nature07128. 18563154

[ref7] Pla J. J.; Tan K. Y.; Dehollain J. P.; Lim W. H.; Morton J. J.; Jamieson D. N.; Dzurak A. S.; Morello A. A single-atom electron spin qubit in silicon. Nature 2012, 489, 541–545. 10.1038/nature11449. 22992519

[ref8] Warner M.; Din S.; Tupitsyn I. S.; Morley G. W.; Stoneham A. M.; Gardener J. A.; Wu Z.; Fisher A. J.; Heutz S.; Kay C. W.; et al. Potential for spin-based information processing in a thin-film molecular semiconductor. Nature 2013, 503, 504–508. 10.1038/nature12597. 24162849

[ref9] Asaad S.; Mourik V.; Joecker B.; Johnson M. A.; Baczewski A. D.; Firgau H. R.; Ma̧dzik M. T.; Schmitt V.; Pla J. J.; Hudson F. E.; et al. Coherent electrical control of a single high-spin nucleus in silicon. Nature 2020, 579, 205–209. 10.1038/s41586-020-2057-7. 32161384

[ref10] de Camargo L. C.; Briganti M.; Santana F. S.; Stinghen D.; Ribeiro R. R.; Nunes G. G.; Soares J. F.; Salvadori E.; Chiesa M.; Benci S.; et al. Exploring the Organometallic Route to Molecular Spin Qubits: the [CpTi(cot)] case. Angew. Chem., Int. Ed. 2021, 60, 2588–2593. 10.1002/anie.202009634. 33051985

[ref11] Atzori M.; Morra E.; Tesi L.; Albino A.; Chiesa M.; Sorace L.; Sessoli R. Quantum Coherence Times Enhancement in Vanadium(IV)-based Potential Molecular Qubits: the Key Role of the Vanadyl Moiety. J. Am. Chem. Soc. 2016, 138, 11234–11244. 10.1021/jacs.6b05574. 27517709

[ref12] Bader K.; Dengler D.; Lenz S.; Endeward B.; Jiang S.-D.; Neugebauer P.; van Slageren J. Room temperature quantum coherence in a potential molecular qubit. Nat. Commun. 2014, 5, 5304 10.1038/ncomms6304. 25328006

[ref13] Schweiger A.; Jeschke G. Principles of Pulse Electron Paramagnetic Resonance; Oxford University Press: Oxford, UK., 2001.

[ref14] Mirzoyan R.; Hadt R. G. The dynamic ligand field of a molecular qubit: Decoherence through spin-phonon coupling. Phys. Chem. Chem. Phys. 2020, 22, 11249–11265. 10.1039/D0CP00852D. 32211668

[ref15] Lunghi A.; Totti F.; Sanvito S.; Sessoli R. Intra-molecular origin of the spin-phonon coupling in slow-relaxing molecular magnets. Chem. Sci. 2017, 8, 6051–6059. 10.1039/C7SC02832F. 28989635 PMC5625570

[ref16] Califano S.; Schettino V.; Neto N. Lattice Dynamics of Molecular Crystals; Springer-Verlag: Berlin, Heidelberg, 1981.

[ref17] Califano S. Vibrational States; Wiley, 1976.

[ref18] Qian K.; Baldoví J. J.; Jiang S.-D.; Gaita-Ariño A.; Zhang Y.-Q.; Overgaard J.; Wang B.-W.; Coronado E.; Gao S. Does the thermal evolution of molecular structures critically affect the magnetic anisotropy?. Chem. Sci. 2015, 6, 4587–4593. 10.1039/C5SC01245G. 29568416 PMC5851079

[ref19] Lawler H. M.; Chang E. K.; Shirley E. L. Dynamical Matrices and Interatomic-Force Constants from Wave-Commensurate Supercells. arXiv preprint cond-mat/0407221, 2004.

[ref20] Wang Y.; Shang S. L.; Fang H.; Liu Z. K.; Chen L. Q. First-principles calculations of lattice dynamics and thermal properties of polar solids. Npj Comput. Mater. 2016, 2, 16006 10.1038/npjcompumats.2016.6.

[ref21] Atzori M.; Tesi L.; Benci S.; Lunghi A.; Righini R.; Taschin A.; Torre R.; Sorace L.; Sessoli R. Spin Dynamics and Low Energy Vibrations: Insights from Vanadyl-Based Potential Molecular Qubits. J. Am. Chem. Soc. 2017, 139, 4338–4341. 10.1021/jacs.7b01266. 28263593

[ref22] Atzori M.; Benci S.; Morra E.; Tesi L.; Chiesa M.; Torre R.; Sorace L.; Sessoli R. Structural Effects on the Spin Dynamics of Potential Molecular Qubits. Inorg. Chem. 2018, 57, 731–740. 10.1021/acs.inorgchem.7b02616. 29280628

[ref23] Yamabayashi T.; Atzori M.; Tesi L.; Cosquer G.; Santanni F.; Boulon M. E.; Morra E.; Benci S.; Torre R.; Chiesa M.; et al. Scaling Up Electronic Spin Qubits into a Three-Dimensional Metal-Organic Framework. J. Am. Chem. Soc. 2018, 140, 12090–12101. 10.1021/jacs.8b06733. 30145887

[ref24] Albino A.; Benci S.; Tesi L.; Atzori M.; Torre R.; Sanvito S.; Sessoli R.; Lunghi A. First-Principles Investigation of Spin-Phonon Coupling in Vanadium-Based Molecular Spin Quantum Bits. Inorg. Chem. 2019, 58, 10260–10268. 10.1021/acs.inorgchem.9b01407. 31343163

[ref25] Lunghi A.; Sanvito S. How do phonons relax molecular spins?. Sci. Adv. 2019, 5, eaax7163 10.1126/sciadv.aax7163. 31598553 PMC6764833

[ref26] Lunghi A.; Sanvito S. The Limit of Spin Lifetime in Solid-State Electronic Spins. J. Phys. Chem. Lett. 2020, 11, 6273–6278. 10.1021/acs.jpclett.0c01681. 32667205

[ref27] Ullah A.; Baldoví J. J.; Gaita-Ariño A.; Coronado E. Insights on the coupling between vibronically active molecular vibrations and lattice phonons in molecular nanomagnets. Dalton Trans. 2021, 50, 11071–11076. 10.1039/D1DT01832A. 34323911

[ref28] Garlatti E.; Tesi L.; Lunghi A.; Atzori M.; Voneshen D. J.; Santini P.; Sanvito S.; Guidi T.; Sessoli R.; Carretta S. Unveiling phonons in a molecular qubit with four-dimensional inelastic neutron scattering and density functional theory. Nat. Commun. 2020, 11, 1751 10.1038/s41467-020-15475-7. 32273510 PMC7145838

[ref29] Taschin A.; Bartolini P.; Tasseva J.; Torre R. THz time-domain spectroscopic investigations of thin films. Measurement 2018, 118, 282–288. 10.1016/j.measurement.2017.05.074.

[ref30] Tasseva J.; Taschin A.; Bartolini P.; Striova J.; Fontana R.; Torre R. Thin layered drawing media probed by THz time-domain spectroscopy. Analyst 2017, 142, 42–47. 10.1039/C6AN02113A. 27900381

[ref31] Taschin A.; Bartolini P.; Tasseva J.; Striova J.; Fontana R.; Riminesi C.; Torre R. Drawing materials studied by THz spectroscopy. Acta Imeko 2017, 6, 12–17. 10.21014/acta_imeko.v6i3.447.

[ref32] Santanni F.; Albino A.; Atzori M.; Ranieri D.; Salvadori E.; Chiesa M.; Lunghi A.; Bencini A.; Sorace L.; Totti F.; et al. Probing Vibrational Symmetry Effects and Nuclear Spin Economy Principles in Molecular Spin Qubits. Inorg. Chem. 2021, 60, 140–151. 10.1021/acs.inorgchem.0c02573. 33305944 PMC7872321

[ref33] Tesi L.; Lunghi A.; Atzori M.; Lucaccini E.; Sorace L.; Totti F.; Sessoli R. Giant spin-phonon bottleneck effects in evaporable vanadyl-based molecules with long spin coherence. Dalton Trans. 2016, 45, 16635–16643. 10.1039/C6DT02559E. 27484897

[ref34] Shuter E.; Rettig S. J.; Orvig C. Oxobis(2,4-pentanedionato)vanadium(IV), a Redetermination. Acta Crystallogr., Sect. C: Cryst. Struct. Commun. 1995, 51, 12–14. 10.1107/S0108270194010462.

[ref35] King M. D.; Korter T. M. Application of London-type dispersion corrections in solid-state density functional theory for predicting the temperature-dependence of crystal structures and terahertz spectra. Cryst. Growth Des. 2011, 11, 2006–2010. 10.1021/cg200211x.

[ref36] Parlinski K. Phonons calculated from first-principles. École thématique de la Société Française de la Neutronique 2011, 12, 161–166. 10.1051/sfn/201112008.

[ref37] Baroni S.; de Gironcoli S.; Dal Corso A.; et al. Phonons and related crystal properties from density-functional perturbation theory. Rev. Mod. Phys. 2001, 73, 515–567. 10.1103/RevModPhys.73.515.

[ref38] VandeVondele J.; Krack M.; Mohamed F.; Parrinello M.; Chassaing T.; Hutter J. QUICKSTEP: Fast and accurate density functional calculations using a mixed Gaussian and plane waves approach. Comput. Phys. Commun. 2005, 167, 103–128. 10.1016/j.cpc.2004.12.014.

[ref39] Kühne T. D.; Iannuzzi M.; Del Ben M.; Rybkin V. V.; Seewald P.; Stein F.; Laino T.; Khaliullin R. Z.; Schütt O.; Schiffmann F.; et al. CP2K: An electronic structure and molecular dynamics software package -Quickstep: Efficient and accurate electronic structure calculations. J. Chem. Phys. 2020, 152, 194103 10.1063/5.0007045. 33687235

[ref40] Goedecker S.; Teter M.; Hutter J. Separable dual-space Gaussian pseudopotentials. Phys. Rev. B 1996, 54, 1703–1710. 10.1103/PhysRevB.54.1703. 9986014

[ref41] Hartwigsen C.; Goedecker S.; Hutter J. Relativistic separable dual-space Gaussian pseudopotentials from H to Rn. Phys. Rev. B 1998, 58, 3641–3662. 10.1103/PhysRevB.58.3641. 9986014

[ref42] Krack M. Pseudopotentials for H to Kr optimized for gradient-corrected exchange-correlation functionals. Theor. Chem. Acc. 2005, 114, 145–152. 10.1007/s00214-005-0655-y.

[ref43] Perdew J. P.; Burke K.; Ernzerhof M. Generalized gradient approximation made simple. Phys. Rev. Lett. 1996, 77, 3865 10.1103/PhysRevLett.77.3865. 10062328

[ref44] Grimme S. Accurate description of van der Waals complexes by density functional theory including empirical corrections. J. Comput. Chem. 2004, 25, 1463–1473. 10.1002/jcc.20078. 15224390

[ref45] Grimme S. Semiempirical GGA-type density functional constructed with a long-range dispersion correction. J. Comput. Chem. 2006, 27, 1787–1799. 10.1002/jcc.20495. 16955487

[ref46] Grimme S.; Antony J.; Ehrlich S.; Krieg H. A consistent and accurate ab initio parametrization of density functional dispersion correction (DFT-D) for the 94 elements H-Pu. J. Chem. Phys. 2010, 132, 154104 10.1063/1.3382344. 20423165

[ref47] Shanno D. F. Conjugate Gradient Methods with Inexact Searches. Math. Oper. Res. 1978, 3, 244–256. 10.1287/moor.3.3.244.

[ref48] Pfrommer B. G.; Cote M.; Louie S. G.; Cohen M. L. Relaxation of Crystals with the Quasi-Newton Method. J. Comput. Phys. 1997, 131, 233–240. 10.1006/jcph.1996.5612.

[ref49] Neese F. Software update: the ORCA program system, version 4.0. Wiley Interdiscip. Rev.: Comput. Mol. Sci. 2018, 8, 1–6. 10.1002/wcms.1327.

[ref50] Adamo C.; Barone V. Toward reliable density functional methods without adjustable parameters: The PBE0 model. J. Chem. Phys. 1999, 110, 6158–6170. 10.1063/1.478522.

[ref51] Lunghi A. Ligand-Field Contributions to Spin-phonon Coupling in a Family of Vanadium Molecular Qubits from Multi-Reference Electronic Structure Theory. arXiv preprint arXiv:1912.04545, 2019.

[ref52] Escalera-Moreno L.; Suaud N.; Gaita-Ariño A.; Coronado E. Determining Key Local Vibrations in the Relaxation of Molecular Spin Qubits and Single-Molecule Magnets. J. Phys. Chem. Lett. 2017, 8, 1695–1700. 10.1021/acs.jpclett.7b00479. 28350165

[ref53] Redfield A. G. Advances in Magnetic and Optical Resonance; Academic Press, 1965; Vol. 1, pp 1–32.

[ref54] Breuer H. P.; Petruccione F. The Theory of Open Quantum Systems; Oxford University Press: New York, 2007.

[ref55] Neto N.; Bellucci L. A new algorithm for rigid body molecular dynamics. Chem. Phys. 2006, 328, 259–268. 10.1016/j.chemphys.2006.07.009.

[ref56] Califano S.; Schettino V. Vibrational relaxation in molecular crystals. Int. Rev. Phys. Chem. 1988, 7, 19–57. 10.1080/01442358809353204.

[ref57] Bini R.; Foggi P.; Salvi P. R.; Schettino V. FTIR study of vibrational relaxation in potassium perchlorate crystal. J. Phys. Chem. A 1990, 94, 6653–6658. 10.1021/j100380a025.

[ref58] Briganti M.; Santanni F.; Tesi L.; Totti F.; Sessoli R.; Lunghi A. A Complete Ab Initio View of Orbach and Raman Spin-Lattice Relaxation in a Dysprosium Coordination Compound. J. Am. Chem. Soc. 2021, 143, 13633–13645. 10.1021/jacs.1c05068. 34465096 PMC8414553

[ref59] Mirzoyan R.; Kazmierczak N. P.; Hadt R. G. Deconvolving Contributions to Decoherence in Molecular Electron Spin Qubits: A Dynamic Ligand Field Approach. Chem. Eur. J. 2021, 27, 9482–9494. 10.1002/chem.202100845. 33855760

[ref60] Shrivastava K. N. Theory of Spin–Lattice Relaxation. Phys. Status Solidi B 1983, 117, 437–458. 10.1002/pssb.2221170202.

[ref61] Iannone F.; Ambrosino F.; Bracco G.; De Rosa M.; Funel A.; Guarnieri G.; Migliori S.; Palombi F.; Ponti G.; Santomauro G.;CRESCO ENEA HPC Clusters: A Working Example of a Multifabric GPFS Spectrum Scale Layout. In Proceedings of the 2019 International Conference on High Performance Computing Simulation (HPCS), 2019; pp 1051–1052.

