# Supplementary material for: Temperature Dependence of Spin–Phonon Coupling in [VO(acac)2]: A Computational and Spectroscopic Study
Source: J Phys Chem C Nanomater Interfaces. 2021 Sep 30;125(40):22100–10. doi: 10.1021/acs.jpcc.1c06916 (PMC8521520; doi:10.1021/acs.jpcc.1c06916)
Supplement: Supplementary file 1 — jp1c06916_si_001.pdf [file jp1c06916_si_001.pdf]

The Temperature Dependence of Spin-Phonon Coupling in $[VO(acac)_2]$: a Computational and Spectroscopic Study

Andrea Albino[a], Stefano Benci[a,b], Matteo Atzori[c], Laura Chelazzi[d], Samuele Ciattini[d], Andrea Taschin[b], Paolo Bartolini[b], Alessandro Lunghi[e], Roberto Righini[b], Renato Torre* [f,b], Federico Totti* [a] and Roberta Sessoli,[a]

[a] *Dipartimento di Chimica "Ugo Schiff" & INSTM RU, Università degli Studi di Firenze, Via della Lastruccia 3, Sesto Fiorentino (FI) 50019, Italy*

[b]*European Laboratory for Non-Linear Spectroscopy (LENS), Università degli Studi di Firenze, Italy*

[c] *Laboratoire National des Champs Magnétiques Intenses (LNCMI), Univ. Grenoble Alpes, INSA Toulouse, Univ. Toulouse Paul Sabatier, EMFL, CNRS, F38043 Grenoble, France.*

[d] *Dipartimento di Chimica "Ugo Schiff" & Center of Crystallography, Università degli Studi di Firenze, Via della Lastruccia 3, Sesto Fiorentino (FI) 50019, Italy*

[e] *School of Physics, CRANN and AMBER, Trinity College, Dublin 2, Ireland*

[f] *Dipartimento di Fisica ed Astronomia, Università degli Studi di Firenze, Via G. Sansone 1, Sesto Fiorentino (FI) 50019, Italy*

Supporting Information

TAB. S 1 Summary of X-ray Crystallographic Data for [VO(acac)$_2$] at 100 K and 300 K

Property	$\mathbf{1_{100K}}$	$\mathbf{1_{300K}}$
empirical formula	$C_{10}H_{14}O_5V$	$C_{10}H_{14}O_5V$
formula weight	265.15	265.15
crystal system	triclinic	triclinic
space group	P-1	P-1
a, Å	7.3005(12)	7.5132(5)
b, Å	8.1172(13)	8.2015(6)
c, Å	11.1782(18)	11.2206(8)
α, deg	72.901(7)	73.174(4)
β, deg	72.282(7)	71.476(4)
γ, deg	67.064(6)	66.718(4)
volume, Å^3	569.37(16)	591.45(8)
Z	2	2
T, K	100(2)	300(2)
ρ (calc), Mg/m^3	1.547	1.489
μ, mm^{-1}	7.351	7.077
θ range, deg	4.24–68.42	4.24–68.32
goodness of fit, Goof	1.191	1.139
R1[a]	0.0563	0.0707
wR2[b]	0.1685	0.1990

[a] $R1 = \Sigma||F_o|-|F_c||/\Sigma|F_o|$,

[b] $wR2 = \left[\Sigma\left[w\left(F_o^2-F_c^2\right)^2\right]/\left[\Sigma w\left(F_o^2\right)^2\right]^{1/2}\right]$,

where $w = 1/\left[\sigma^2\left(F_o^2\right)+(aP)^2+bP\right]$,

and $P = \left[\max\left(F_o^2,0\right)+2F_c^2\right]/3$.

Convergence test: DFT Parameters vs. PW cutoff

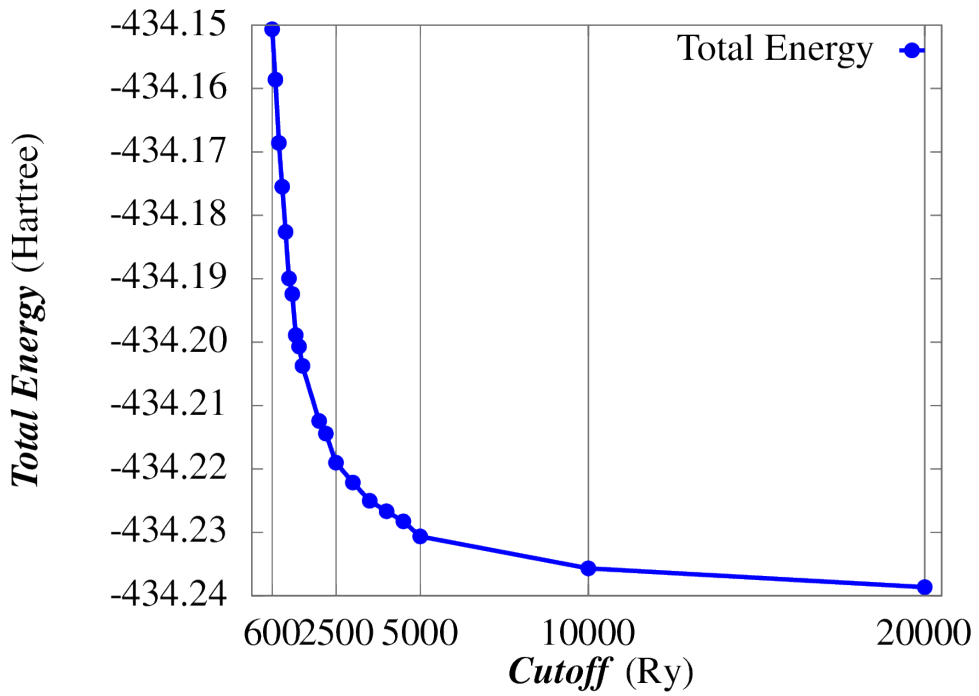

FIG. S 1 Plot of DFT Total Energy calculation results as a function of plane waves cutoff.

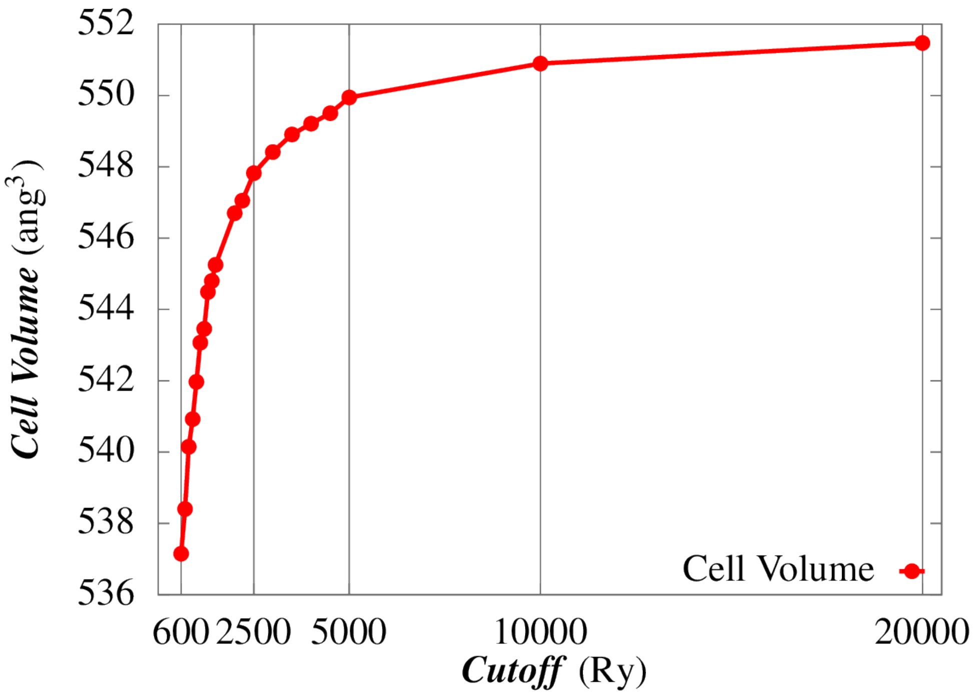

FIG. S 2 Plot of DFT Cell Volume calculation results as a function of plane waves cutoff.

VO(acac)$_2$ Cartesian Derivatives Comparison

TAB. S 2 The derivatives $\dfrac{\partial g_{XX}}{\partial r_\alpha}$ of the diagonal g_{XX} element of the Landè factor are displayed for the Vanadium(IV) metal center and for the five peripheral Oxigen atoms. α index runs over the $3N$ cartesian coordinates r and N is the number of atoms in the molecule.

α	r_α	0K cell opt	100K geo opt	300K geo opt
1	x_V	-1.46203088E-02	-1.21445060E-02	-1.24181770E-02
2	y_V	-2.76838131E-02	-2.74094734E-02	-2.87556536E-02
3	z_V	2.89104618E-02	3.22515853E-02	3.09840161E-02
4	x_O^{acac}	-1.47276092E-02	-1.45998057E-02	-3.69157759E-03
5	y_O^{acac}	-1.38264429E-03	-1.40079285E-03	-3.85635463E-03
6	z_O^{acac}	6.90926192E-03	6.49499521E-03	-1.70013849E-02
7	x_O^{acac}	-4.17997874E-03	-3.37451557E-03	-8.73978715E-03
8	y_O^{acac}	-3.75643442E-03	-4.22891276E-03	5.78277512E-03
9	z_O^{acac}	-1.66153368E-02	-1.73409153E-02	-3.46001441E-04
10	x_O^{acac}	-7.58819887E-03	-9.13951546E-03	1.00584226E-02
11	y_O^{acac}	6.16217591E-03	5.65277738E-03	1.76612455E-02
12	z_O^{acac}	-4.80698800E-04	-3.53019772E-04	-1.99710838E-02
13	x_O^{acac}	1.06107909E-02	1.00607947E-02	-1.46265775E-02
14	y_O^{acac}	1.71720870E-02	1.74093265E-02	-1.13889459E-03
15	z_O^{acac}	-1.91540383E-02	-1.99739151E-02	6.97050570E-03
16	$x_O^{vanadyl}$	2.86301915E-02	2.74688937E-02	2.78202221E-02
17	$y_O^{vanadyl}$	4.29155584E-03	5.04433736E-03	5.06365532E-03
18	$z_O^{vanadyl}$	-3.01737781E-03	-3.89244477E-03	-3.57094430E-03

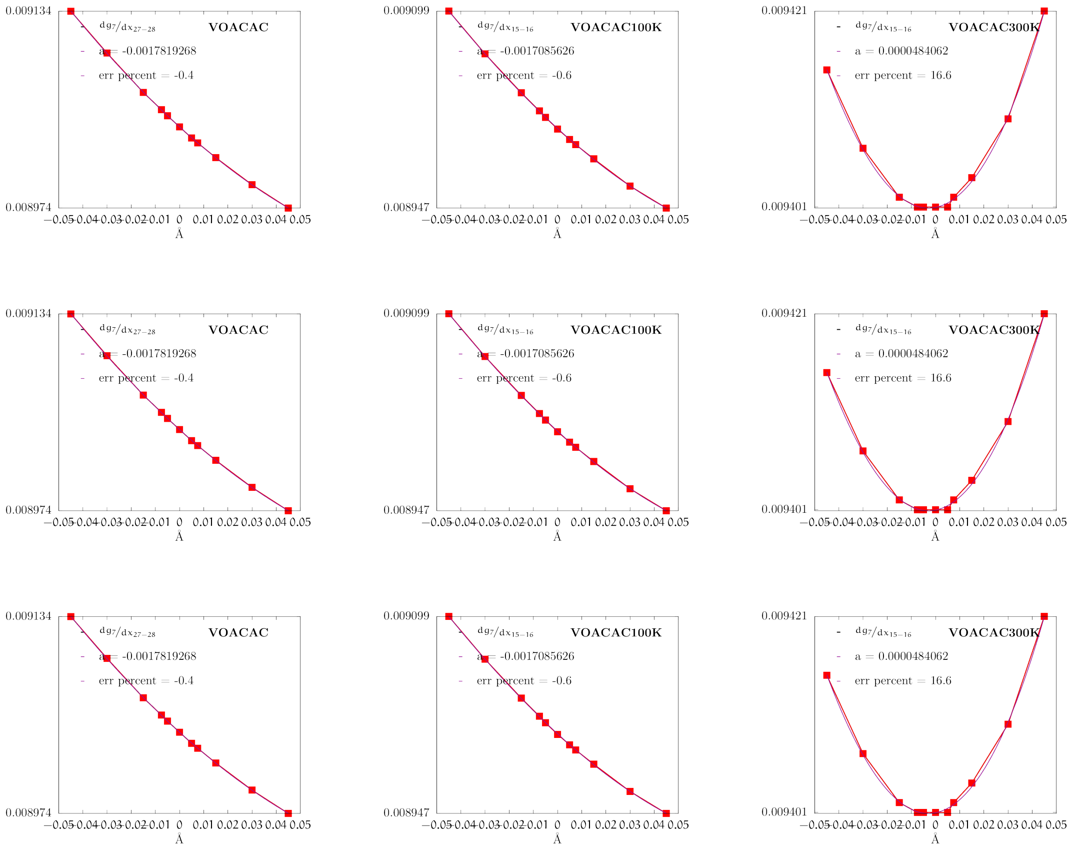

FIG. S 3 Derivatives may vary (not dramatically) between 0K-100K-300K structures.

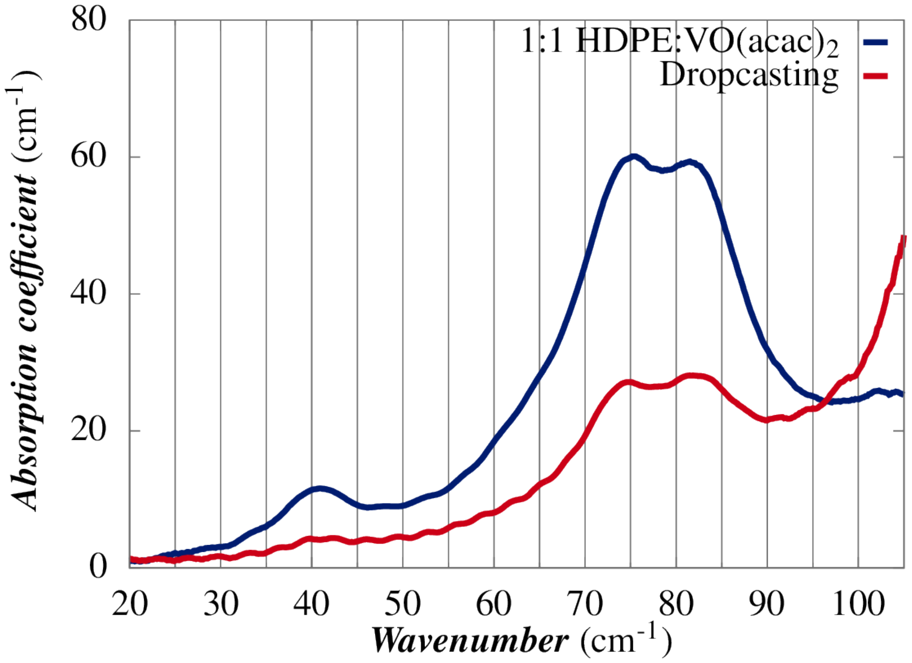

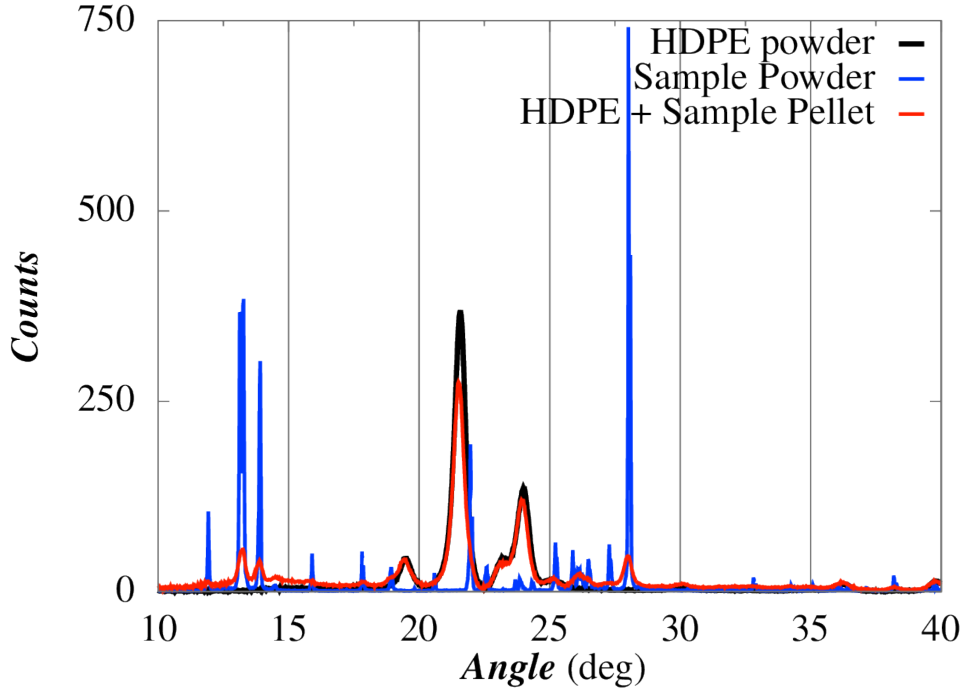

FIG. S 4 Top: comparison of ($\mathbf{1_a}$) and ($\mathbf{1_b}$). No significant effects are originated by the preparation method of each sample, confirming that the applied pressure, necessary to make the pellet, does not change the structure of [$VO(acac)_2$]. Bottom: powders X-Ray diffraction analysis. Comparison among pure HDPE powder (black line), pure [$VO(acac)_2$] powder (green line) and the pellet of the sample dispersed in HDPE.

TAB. S 3 Full spectral data of $\mathbf{1}_{\mathbf{0K,cellopt}}$, $\mathbf{1}_{\mathbf{100K,opt}}$, $\mathbf{1}_{\mathbf{300K,opt}}$. ω is the mode frequency, *i* shows the presence (RA) or absence (RA) of inversion point in the mode simmetry, IR int. represents the IR intensity and Sp-Ph represents the spin-phonon coupling coefficient magnitude.

	$\mathbf{1}_{\mathbf{0K,cellopt}}$					$\mathbf{1}_{\mathbf{100K,opt}}$					$\mathbf{1}_{\mathbf{300K,opt}}$			
	ω (cm^{-1})	*i*	IR int.	Sp-Ph		ω (cm^{-1})	*i*	IR int.	Sp-Ph		ω (cm^{-1})	*i*	IR int.	Sp-Ph
4	43.313	RA	8.04759e-08	1.130E-7	4	42.411	IR	0.109243	3.905E-7	4	38.137	IR	0.12052	3.787E-7
5	50.872	RA	3.31624e-08	1.648E-7	5	46.320	RA	4.77063e-09	1.430E-7	5	40.497	RA	1.8248e-08	2.427E-8
6	55.323	IR	0.111876	2.331E-7	6	48.492	RA	2.55834e-08	3.037E-7	6	43.222	RA	4.52691e-08	3.545E-7
7	63.349	RA	9.19853e-08	6.260E-7	7	57.788	RA	7.80443e-09	7.608E-7	7	53.353	RA	1.20301e-08	5.580E-7
8	73.267	IR	0.183217	4.399E-7	8	66.991	RA	6.69308e-09	3.717E-7	8	61.592	RA	1.80185e-08	3.906E-7
9	75.290	RA	5.41484e-08	2.357E-7	9	69.518	IR	0.137836	8.549E-7	9	64.171	IR	0.15482	8.199E-7
10	81.218	RA	2.52218e-07	3.927E-7	10	76.013	IR	0.0503467	4.069E-7	10	69.467	IR	0.0260264	2.448E-7
11	83.461	IR	0.0793074	2.453E-7	11	79.998	RA	2.59466e-09	9.584E-8	11	73.577	RA	4.64896e-09	1.711E-7
12	98.285	RA	6.83067e-07	4.581E-7	12	94.864	IR	0.310476	6.695E-7	12	86.907	IR	0.3602	6.289E-7
13	99.240	IR	0.160532	3.330E-7	13	100.610	RA	7.95437e-08	9.038E-7	13	89.691	RA	5.7009e-08	6.472E-7
14	111.392	RA	1.4703e-07	2.764E-7	14	104.128	RA	4.84387e-09	4.613E-7	14	96.560	RA	2.50481e-09	4.950E-7
15	112.346	IR	0.142351	5.968E-7	15	108.685	IR	0.027687	3.620E-7	15	102.072	IR	0.0390919	2.255E-7
16	114.618	RA	1.68674e-07	2.050E-7	16	110.094	RA	1.1741e-09	1.703E-7	16	102.096	RA	4.38438e-08	3.003E-7
17	116.558	IR	0.098746	2.171E-7	17	116.262	IR	0.122146	4.334E-7	17	106.960	IR	0.0981516	4.870E-7
18	125.108	IR	0.0340334	2.842E-7	18	120.492	IR	0.118287	1.231E-7	18	113.787	IR	0.101774	6.313E-8
19	135.847	RA	8.65943e-09	2.568E-7	19	124.879	RA	2.74591e-08	8.979E-8	19	115.077	IR	0.0537505	4.863E-7
20	145.745	IR	0.0843109	8.805E-8	20	125.933	IR	0.0311634	2.795E-7	20	115.669	RA	7.66743e-09	6.292E-8
21	147.043	RA	2.57304e-07	4.963E-8	21	131.801	RA	1.93044e-09	1.043E-7	21	123.471	RA	4.91208e-09	1.616E-7
22	153.477	RA	1.41611e-08	9.594E-8	22	146.619	RA	4.8419e-10	8.473E-8	22	132.512	RA	4.1744e-10	1.081E-7
23	154.161	IR	0.00478915	3.896E-8	23	147.894	IR	0.00022307	1.059E-7	23	137.701	IR	0.00659183	1.032E-7
24	157.023	RA	1.67448e-09	3.143E-7	24	152.115	RA	3.8858e-10	4.251E-8	24	142.470	RA	9.00518e-09	9.581E-8
25	164.811	IR	0.0168672	1.544E-7	25	156.494	RA	3.46825e-08	3.604E-7	25	146.251	IR	0.0261749	1.275E-7
26	170.864	IR	0.0228392	8.523E-8	26	156.901	IR	0.0155736	9.248E-8	26	147.674	RA	1.74798e-08	3.167E-7
27	172.283	RA	5.20073e-08	7.640E-8	27	159.188	IR	0.0501272	9.521E-8	27	150.326	IR	0.0154465	1.132E-7
28	176.786	RA	1.37239e-09	7.966E-8	28	171.287	RA	4.79582e-10	5.244E-8	28	152.845	RA	3.60875e-09	6.060E-8
29	182.392	RA	4.246e-09	1.085E-7	29	178.512	IR	0.0103048	5.849E-8	29	166.038	IR	0.00195887	4.370E-8
30	191.142	IR	0.0407331	2.090E-7	30	179.447	RA	6.50843e-09	5.093E-8	30	170.613	RA	3.40344e-11	4.378E-8
31	191.914	IR	0.00704056	2.054E-7	31	184.578	IR	0.00895319	2.577E-7	31	176.841	IR	0.0102557	1.358E-7
32	197.706	IR	0.0364393	3.643E-7	32	190.518	RA	3.93853e-07	5.654E-7	32	184.088	RA	7.13285e-10	4.614E-7
33	198.410	RA	5.03695e-07	3.365E-7	33	190.580	IR	0.0672073	3.086E-7	33	185.233	IR	0.0500268	3.206E-7
34	214.610	RA	4.10336e-09	1.484E-6	34	203.046	IR	0.0078023	2.395E-7	34	191.400	RA	3.11997e-09	1.035E-7
35	221.435	IR	0.0289395	9.542E-7	35	205.520	RA	2.92579e-09	3.848E-7	35	195.487	IR	0.0208491	3.419E-7
36	223.183	RA	1.03994e-08	9.031E-7	36	210.897	RA	3.72967e-09	6.500E-7	36	202.043	RA	9.46518e-10	3.840E-7
37	226.889	IR	0.0999659	1.988E-6	37	216.045	RA	1.6324e-09	1.729E-6	37	209.752	RA	9.01762e-10	7.126E-7
38	232.051	RA	2.73578e-08	1.202E-6	38	218.329	IR	0.0771787	2.982E-6	38	216.655	IR	0.0611255	2.870E-6
39	235.632	IR	0.0110861	7.643E-7	39	225.085	RA	5.69773e-10	4.600E-7	39	216.774	RA	7.77778e-06	1.797E-6
40	238.610	IR	0.0508659	2.508E-6	40	233.321	IR	0.03545	2.727E-6	40	225.294	IR	0.0285509	2.083E-7
41	242.644	RA	6.13992e-10	1.443E-6	41	237.690	IR	0.0372088	7.706E-7	41	232.769	IR	0.0540075	3.343E-6
42	248.507	RA	3.03884e-09	8.837E-7	42	237.990	RA	3.78922e-09	2.896E-6	42	234.876	RA	8.61177e-08	3.177E-6
43	261.756	RA	1.94294e-09	4.008E-7	43	259.801	RA	1.14391e-08	3.058E-7	43	256.280	IR	0.0445536	6.833E-7
44	262.168	IR	0.00241313	2.946E-7	44	259.858	IR	0.0324196	4.775E-7	44	257.025	RA	2.37159e-09	2.938E-7
45	266.121	IR	0.129181	1.806E-6	45	260.527	IR	0.0759373	1.145E-6	45	257.512	IR	0.00407065	2.797E-7
46	267.209	IR	0.0383872	5.459E-7	46	262.110	RA	6.45148e-09	1.669E-6	46	258.013	RA	2.78966e-08	6.138E-7
47	267.646	RA	6.10078e-07	1.540E-6	47	264.081	IR	0.0381919	5.743E-7	47	262.248	IR	0.0661621	1.029E-6
48	278.568	RA	1.07569e-09	1.004E-7	48	264.431	RA	4.61031e-08	7.493E-8	48	262.352	RA	2.25698e-07	9.585E-7
49	299.488	IR	0.0951757	1.396E-6	49	291.124	IR	0.124052	1.694E-6	49	286.495	IR	0.137506	2.013E-6
50	307.599	RA	2.21386e-09	1.551E-6	50	298.739	RA	2.45054e-10	1.717E-6	50	292.978	RA	2.10202e-10	1.988E-6
51	362.124	IR	0.345906	1.573E-6	51	360.098	IR	0.343246	1.510E-6	51	360.154	IR	0.331754	1.527E-6
52	365.808	RA	5.98087e-09	2.254E-6	52	363.302	RA	9.96037e-09	2.356E-6	52	362.357	RA	1.98123e-08	2.564E-6
53	370.550	IR	0.0236267	2.889E-6	53	367.026	IR	0.00930416	3.132E-6	53	365.217	IR	0.010304	3.259E-6
54	372.937	RA	2.78906e-10	1.992E-6	54	369.740	RA	6.7007e-10	1.940E-6	54	368.039	RA	6.24529e-10	1.864E-6
55	404.792	IR	0.00638653	4.832E-8	55	404.274	IR	0.00427885	3.692E-8	55	404.406	IR	0.00387183	3.153E-8
56	405.704	RA	1.40575e-08	1.382E-8	56	404.822	RA	1.56788e-08	3.494E-9	56	404.502	RA	2.68418e-07	4.610E-9
57	420.684	IR	0.118925	6.350E-7	57	419.717	IR	0.115886	5.391E-7	57	419.626	IR	0.111054	5.464E-7
58	422.250	RA	7.24816e-08	6.958E-7	58	420.367	RA	4.66749e-07	6.222E-7	58	420.259	RA	9.17834e-08	6.117E-7
59	460.382	IR	0.103504	2.155E-6	59	455.743	IR	0.0885806	2.161E-6	59	454.217	IR	0.0807651	2.161E-6
60	464.761	RA	5.77817e-10	2.299E-6	60	460.013	RA	3.79541e-09	2.289E-6	60	458.066	RA	2.05066e-10	2.287E-6
61	474.153	IR	1.20604	3.470E-6	61	468.580	IR	1.25879	3.621E-6	61	466.492	IR	1.23675	3.707E-6
62	483.878	RA	2.18143e-09	3.677E-6	62	479.723	RA	6.18163e-10	3.840E-6	62	476.911	RA	7.978e-09	3.924E-6
63	536.986	IR	0.017771	7.517E-8	63	540.858	IR	0.0130267	7.519E-8	63	541.228	IR	0.0115712	6.476E-8
64	543.112	RA	4.91258e-10	7.022E-8	64	544.275	RA	3.61066e-11	5.876E-8	64	543.919	RA	1.78257e-10	5.375E-8
65	548.183	IR	0.00463221	5.654E-9	65	547.075	IR	0.00895865	4.307E-9	65	547.140	IR	0.0072697	3.158E-9
66	548.972	RA	1.48482e-09	2.555E-9	66	549.520	RA	5.42449e-11	4.758E-9	66	549.207	RA	3.44394e-11	1.967E-9
67	575.367	RA	1.61954e-09	5.960E-9	67	571.414	IR	0.00421952	1.323E-8	67	569.910	IR	0.00251436	7.838E-9
68	575.913	IR	0.00139236	3.853E-9	68	572.319	RA	7.59549e-10	1.295E-8	68	570.358	RA	1.02047e-09	7.796E-9
69	604.371	IR	0.255618	5.990E-7	69	600.870	IR	0.216155	5.797E-7	69	598.911	IR	0.215597	5.974E-7
70	606.982	RA	1.19296e-10	6.019E-7	70	602.671	RA	3.75203e-09	5.942E-7	70	600.666	RA	5.72882e-09	6.102E-7
71	648.631	IR	0.0193893	1.245E-7	71	648.634	IR	0.0299205	1.481E-7	71	649.770	IR	0.017256	1.014E-7
72	650.156	RA	1.32074e-08	1.373E-7	72	650.569	IR	0.0340785	2.248E-7	72	651.041	IR	0.0434422	2.683E-7

73	650.513	IR	0.0427074	1.983E-7	73	652.863	RA	7.63423e-10	3.026E-7	73	652.641	RA	5.95104e-10	3.135E-7
74	653.981	RA	6.4949e-09	1.559E-7	74	653.845	RA	5.11941e-11	2.672E-8	74	653.602	RA	2.83636e-10	1.720E-8
75	678.241	RA	7.84943e-11	7.079E-7	75	673.688	RA	5.40983e-09	6.370E-7	75	672.178	RA	1.43499e-08	6.783E-7
76	680.556	IR	0.255485	6.022E-7	76	674.832	IR	0.210839	5.123E-7	76	673.748	IR	0.225122	5.553E-7
77	684.168	IR	0.011226	3.055E-7	77	681.210	IR	0.0496708	3.769E-7	77	678.588	IR	0.0267721	3.368E-7
78	685.792	RA	2.04802e-09	2.849E-7	78	682.162	RA	1.41083e-09	3.396E-7	78	679.614	RA	9.32955e-11	3.006E-7
79	806.882	IR	0.0724555	3.436E-8	79	801.662	IR	0.0687535	3.930E-8	79	797.634	IR	0.0656518	3.650E-8
80	807.874	RA	3.29677e-06	1.363E-8	80	802.210	RA	4.0551e-08	1.649E-8	80	797.813	RA	5.29758e-07	1.546E-8
81	824.896	IR	0.167381	3.563E-8	81	812.944	IR	0.137611	2.891E-8	81	810.675	IR	0.126061	2.822E-8
82	826.085	RA	4.07331e-07	3.382E-8	82	814.401	RA	2.95462e-08	1.993E-8	82	811.541	RA	2.00638e-08	2.367E-8
83	933.082	IR	0.04976	1.904E-8	83	932.924	RA	1.6486e-07	1.039E-7	83	931.214	RA	2.04377e-08	9.323E-8
84	934.233	RA	1.80486e-05	8.996E-8	84	933.007	IR	0.0250772	8.661E-8	84	931.278	IR	0.0163319	5.245E-8
85	934.301	IR	0.0889706	2.901E-7	85	934.272	IR	0.115405	1.866E-7	85	932.763	IR	0.117445	2.284E-7
86	935.405	RA	5.55405e-08	1.772E-7	86	935.338	RA	2.02795e-09	1.037E-7	86	933.549	RA	2.5826e-09	1.411E-7
87	941.657	IR	0.100162	1.959E-7	87	935.746	IR	0.0493726	2.277E-7	87	935.568	IR	0.051768	2.337E-7
88	941.810	RA	1.77867e-07	1.908E-7	88	937.822	RA	5.90178e-11	2.470E-7	88	937.058	RA	1.2154e-09	1.911E-7
89	945.553	RA	1.44078e-07	2.848E-7	89	939.732	RA	2.16701e-08	2.535E-7	89	938.673	RA	5.29647e-08	2.632E-7
90	945.826	IR	0.102302	1.893E-7	90	940.038	IR	0.0999759	1.738E-7	90	939.130	IR	0.0900945	1.526E-7
91	997.163	RA	1.50785e-08	8.506E-6	91	1000.742	RA	5.26222e-10	8.319E-6	91	999.858	RA	1.72929e-08	8.338E-6
92	997.631	IR	0.0399555	1.295E-6	92	1001.872	IR	0.191185	3.224E-6	92	1001.160	IR	0.0365619	1.109E-6
93	1000.957	IR	0.796356	8.655E-6	93	1002.603	IR	0.610095	3.181E-6	93	1002.844	IR	0.796057	7.384E-6
94	1003.876	RA	7.14566e-09	7.658E-7	94	1005.739	IR	0.171442	3.310E-6	94	1005.060	IR	0.0531896	1.021E-6
95	1007.821	IR	0.112295	1.266E-7	95	1006.923	RA	8.22972e-11	3.859E-7	95	1006.004	RA	4.35763e-11	3.096E-7
96	1013.269	RA	4.36423e-07	3.356E-8	96	1011.082	RA	5.43189e-10	1.110E-7	96	1010.913	RA	6.82764e-10	2.124E-7
97	1014.056	IR	0.107557	1.993E-7	97	1011.997	IR	0.0412148	1.547E-8	97	1011.729	IR	0.0665485	6.118E-8
98	1014.784	RA	1.38735e-07	3.304E-7	98	1013.710	RA	3.47201e-10	8.650E-8	98	1012.239	RA	9.12332e-09	1.472E-7
99	1015.711	IR	0.0346709	2.947E-8	99	1014.332	RA	6.11686e-09	5.616E-7	99	1014.151	IR	0.0385216	6.333E-7
100	1016.549	RA	5.73844e-09	5.885E-7	100	1014.833	IR	0.0431644	4.908E-7	100	1014.163	RA	1.62183e-07	4.780E-7
101	1018.734	RA	0.00483498	2.417E-7	101	1016.008	IR	0.0372231	2.748E-7	101	1015.589	IR	0.0443421	2.862E-7
102	1019.930	RA	3.38397e-08	2.926E-7	102	1017.671	RA	2.21068e-10	6.963E-7	102	1017.017	RA	1.31692e-09	8.189E-7
103	1022.947	IR	0.0821304	9.367E-8	103	1019.948	IR	0.133537	1.052E-7	103	1018.931	IR	0.110751	1.386E-7
104	1023.574	RA	6.96194e-08	4.093E-8	104	1020.294	RA	1.82752e-08	1.475E-7	104	1018.935	RA	4.02648e-05	7.516E-8
105	1027.274	IR	0.0534552	1.849E-7	105	1022.683	IR	0.0338383	1.942E-7	105	1021.913	IR	0.0438113	1.870E-7
106	1027.729	RA	1.8879e-07	2.396E-7	106	1024.485	RA	4.42101e-10	4.741E-7	106	1023.443	RA	1.96321e-10	4.532E-7
107	1029.187	IR	0.047155	4.214E-8	107	1027.409	IR	0.0237977	1.354E-7	107	1027.004	IR	0.0285083	7.274E-8
108	1032.462	RA	3.41338e-09	4.497E-8	108	1029.281	RA	4.32922e-10	1.273E-7	108	1028.590	RA	1.16858e-09	4.104E-8
109	1187.200	IR	0.00686606	1.783E-8	109	1182.955	IR	0.0066375	1.498E-8	109	1181.911	IR	0.00614484	1.548E-8
110	1188.392	RA	2.76196e-09	8.231E-9	110	1183.921	RA	4.50919e-11	6.070E-9	110	1182.726	RA	5.71022e-11	7.109E-9
111	1194.633	IR	0.0203137	4.122E-8	111	1187.669	IR	0.0239093	4.556E-8	111	1186.775	IR	0.0232716	4.288E-8
112	1195.105	RA	9.40222e-07	4.381E-8	112	1188.217	RA	1.82926e-09	4.907E-8	112	1187.336	RA	3.01665e-10	4.542E-8
113	1288.207	RA	1.52309e-08	3.005E-8	113	1281.433	RA	1.86738e-10	3.016E-8	113	1279.819	RA	5.59243e-10	2.931E-8
114	1290.855	IR	0.30865	2.654E-8	114	1283.654	IR	0.302599	2.300E-8	114	1282.180	IR	0.302961	2.315E-8
115	1294.384	RA	2.62571e-09	8.713E-9	115	1287.009	RA	1.21751e-10	1.087E-8	115	1285.911	RA	3.29892e-11	1.168E-8
116	1297.518	IR	0.0144768	9.810E-9	116	1290.885	IR	0.0210566	1.320E-8	116	1288.952	IR	0.0204898	1.313E-8
117	1346.097	RA	3.64855e-08	2.579E-9	117	1348.487	RA	2.12425e-10	4.888E-9	117	1349.334	RA	3.48816e-09	4.287E-9
118	1351.558	IR	0.388836	3.260E-8	118	1350.103	IR	0.468375	4.751E-8	118	1350.512	IR	0.436124	4.416E-8
119	1360.046	RA	8.34929e-08	1.319E-8	119	1357.440	IR	0.0237268	1.604E-8	119	1357.443	IR	0.0223553	1.404E-8
120	1360.716	IR	0.0292593	1.929E-8	120	1358.480	RA	4.20755e-10	1.563E-8	120	1357.930	RA	1.22209e-09	1.296E-8
121	1362.377	IR	0.0365405	2.946E-9	121	1361.034	IR	0.0557964	9.472E-9	121	1360.546	IR	0.0371884	9.293E-9
122	1370.302	RA	3.7814e-10	9.134E-9	122	1365.396	RA	1.45129e-10	8.974E-9	122	1365.585	RA	1.79737e-10	7.479E-9
123	1372.767	RA	4.45359e-09	3.334E-9	123	1368.694	IR	0.0207626	7.809E-9	123	1369.221	RA	1.27339e-09	2.142E-8
124	1374.784	IR	0.0398089	8.164E-9	124	1369.515	RA	3.27039e-10	2.663E-8	124	1369.386	IR	0.0212602	8.359E-9
125	1378.058	RA	4.90594e-08	3.217E-8	125	1372.610	IR	0.0424665	1.421E-8	125	1373.265	IR	0.0499414	1.886E-8
126	1381.429	IR	0.0896561	6.519E-9	126	1374.567	RA	2.07373e-10	6.853E-9	126	1373.494	RA	1.21373e-08	1.121E-8
127	1384.681	RA	2.50782e-08	2.672E-7	127	1379.334	RA	5.63455e-11	2.726E-7	127	1380.122	RA	9.58987e-10	2.867E-7
128	1386.276	IR	0.622979	2.276E-7	128	1383.494	IR	0.537896	2.167E-7	128	1383.334	IR	0.535913	2.255E-7
129	1411.738	IR	0.515631	3.299E-8	129	1414.155	IR	0.625517	2.583E-8	129	1415.400	IR	0.635917	2.806E-8
130	1422.711	RA	7.73378e-09	2.223E-8	130	1421.912	RA	4.00316e-10	7.905E-9	130	1423.182	RA	5.67202e-10	5.688E-9
131	1427.750	IR	0.117887	1.028E-8	131	1422.988	IR	0.0743813	1.090E-8	131	1423.604	IR	0.0228707	7.665E-9
132	1428.689	IR	0.160107	2.018E-8	132	1426.402	RA	4.37814e-11	2.311E-8	132	1425.176	RA	6.86207e-11	2.660E-8
133	1430.373	RA	1.98635e-09	1.999E-8	133	1429.381	IR	0.109937	1.238E-8	133	1428.631	IR	0.100581	1.211E-8
134	1436.946	IR	0.0192226	6.545E-9	134	1438.142	IR	0.0329663	3.172E-9	134	1437.660	RA	1.2147e-09	1.556E-8
135	1439.499	RA	1.13278e-09	1.880E-8	135	1440.524	RA	3.33782e-11	1.457E-8	135	1437.957	IR	0.032662	3.358E-9
136	1446.695	RA	7.51618e-09	2.238E-9	136	1443.750	RA	2.07075e-10	3.991E-9	136	1442.552	RA	7.99518e-10	5.104E-9
137	1447.346	IR	0.0247034	1.736E-8	137	1444.558	IR	0.0289408	1.076E-8	137	1443.724	IR	0.038336	8.271E-9
138	1451.251	RA	2.4592e-09	1.583E-9	138	1448.016	IR	0.0524794	1.579E-8	138	1447.087	IR	0.0403361	1.386E-8
139	1455.902	IR	0.0545879	2.682E-9	139	1449.135	RA	4.24519e-10	1.806E-9	139	1447.935	RA	2.3485e-11	1.893E-9
140	1457.802	RA	1.01536e-08	1.096E-8	140	1452.422	RA	5.68649e-11	1.310E-8	140	1451.571	RA	4.60836e-11	7.330E-9
141	1459.155	IR	0.0247834	4.499E-8	141	1456.951	IR	0.0203876	2.820E-8	141	1456.499	IR	0.0161374	5.389E-8
142	1462.833	IR	0.0581313	1.943E-8	142	1458.962	RA	1.1648e-10	7.596E-9	142	1456.630	RA	1.99182e-10	7.488E-9
143	1463.086	RA	2.70235e-06	6.790E-9	143	1459.939	IR	0.0363434	1.733E-8	143	1457.804	IR	0.0331246	2.391E-9
144	1469.852	RA	1.40281e-09	5.166E-8	144	1462.136	RA	1.41314e-10	4.178E-8	144	1461.027	RA	1.0522e-10	4.853E-8
145	1523.527	RA	3.33511e-07	8.469E-8	145	1517.201	RA	8.15059e-11	7.183E-8	145	1517.858	RA	2.34833e-09	7.171E-8
146	1524.718	IR	1.62786	8.583E-8	146	1521.722	IR	1.56251	7.710E-8	146	1522.833	IR	1.58104	7.509E-8
147	1535.695	RA	9.4273e-09	1.223E-7	147	1530.226	RA	7.98863e-10	1.167E-7	147	1530.515	RA	1.13845e-10	9.720E-8
148	1544.567	IR	0.026093	2.746E-8	148	1538.172	IR	0.0538025	2.809E-8	148	1537.143	IR	0.0405355	2.055E-8
149	1549.952	IR	1.82777	5.819E-8	149	1541.411	IR	1.74561	6.296E-8	149	1540.714	IR	1.68293	6.892E-8
150	1554.565	RA	8.259e-09	1.134E-7	150	1548.800	RA	1.53348e-11	8.716E-8	150	1547.669	RA	3.11708e-10	8.699E-8
151	1561.453	RA	2.49634e-08	1.986E-7	151	1552.768	RA	2.05354e-10	2.532E-7	151	1552.427	RA	1.19596e-11	2.825E-7

152	1563.036	IR	0.0467863	3.438E-7	152	1560.816	IR	0.0547118	3.569E-7	152	1560.852	IR	0.0525904	3.713E-7
153	2984.609	RA	5.82463e-07	1.576E-010	153	2987.477	IR	0.0314729	6.862E-011	153	2984.984	IR	0.02508	1.074E-010
154	2984.836	IR	0.0373476	8.844E-011	154	2987.614	RA	5.40876e-09	1.583E-010	154	2986.748	RA	4.31484e-12	2.470E-010
155	2987.178	IR	0.0354518	4.113E-010	155	2989.751	IR	0.027779	3.763E-010	155	2988.618	RA	1.52361e-11	2.115E-010
156	2987.338	RA	8.05954e-08	2.855E-010	156	2990.494	RA	3.71615e-10	2.708E-010	156	2989.806	IR	0.0231198	3.906E-010
157	3003.416	RA	1.88664e-11	1.629E-010	157	2994.141	RA	8.65443e-11	1.757E-010	157	2990.776	RA	2.16912e-12	1.612E-010
158	3008.649	IR	0.00602421	1.575E-010	158	2996.398	IR	0.00809659	1.424E-010	158	2992.997	IR	0.00761636	1.279E-010
159	3022.784	IR	0.0214359	6.555E-011	159	3007.206	IR	0.0127427	9.826E-011	159	3006.536	IR	0.0134931	1.335E-010
160	3022.798	RA	9.88563e-05	1.228E-010	160	3007.336	RA	1.51399e-09	9.204E-011	160	3006.651	RA	1.23586e-11	1.354E-010
161	3077.354	IR	0.00816996	2.033E-010	161	3071.540	RA	4.17023e-12	7.330E-011	161	3068.363	RA	1.07272e-10	7.217E-011
162	3078.320	RA	2.00665e-09	1.258E-010	162	3071.975	IR	0.0101648	1.379E-010	162	3068.828	IR	0.00968949	1.361E-010
163	3079.200	RA	6.99323e-09	3.136E-011	163	3074.971	RA	1.3102e-11	1.636E-011	163	3072.223	RA	1.89979e-11	2.025E-011
164	3080.333	IR	0.0285368	1.275E-010	164	3076.782	IR	0.03712	1.268E-010	164	3073.876	IR	0.0284894	1.303E-010
165	3098.381	RA	2.96741e-08	2.515E-010	165	3088.761	RA	1.14497e-07	1.116E-010	165	3085.901	RA	9.87027e-10	1.066E-010
166	3098.432	IR	0.00609919	2.007E-010	166	3088.770	IR	0.0123451	1.479E-010	166	3085.974	IR	0.0107032	7.115E-011
167	3104.466	IR	0.00484697	1.333E-010	167	3094.222	IR	0.00280017	1.627E-010	167	3089.976	IR	0.00414112	1.318E-010
168	3104.715	RA	1.69719e-07	1.165E-010	168	3094.315	RA	7.55871e-10	1.488E-010	168	3090.099	RA	4.13466e-09	1.151E-010
169	3132.334	RA	2.77076e-07	1.886E-011	169	3122.378	RA	2.44764e-09	2.255E-011	169	3119.604	RA	2.05927e-10	2.091E-011
170	3132.517	IR	0.0063204	3.671E-012	170	3122.530	IR	0.0053411	5.737E-012	170	3119.770	IR	0.00467375	4.427E-012
171	3134.004	RA	1.42754e-08	1.184E-011	171	3124.077	RA	4.12205e-10	7.871E-012	171	3122.671	RA	6.22569e-10	9.876E-012
172	3134.449	IR	0.00530281	3.087E-011	172	3124.339	IR	0.0041218	3.117E-011	172	3122.865	IR	0.00381488	2.561E-011
173	3153.093	RA	3.47523e-08	3.727E-011	173	3139.376	RA	1.94498e-10	2.989E-010	173	3134.794	RA	2.5466e-10	1.086E-010
174	3153.765	IR	0.0142862	4.449E-011	174	3140.031	IR	0.0153815	2.744E-010	174	3135.445	IR	0.0137782	1.183E-010
175	3161.440	RA	6.17076e-07	8.247E-011	175	3148.231	RA	8.64978e-09	1.681E-010	175	3142.252	RA	6.51733e-11	1.774E-010
176	3161.592	IR	0.00200373	9.412E-011	176	3148.297	IR	0.00173749	1.864E-010	176	3142.327	IR	0.00190462	1.869E-010
177	3178.047	RA	1.88405e-07	2.996E-010	177	3170.090	RA	1.90442e-07	3.189E-010	177	3171.188	RA	2.64077e-08	2.298E-010
178	3178.086	IR	0.0165278	1.997E-010	178	3170.092	IR	0.00314695	2.587E-010	178	3171.214	IR	0.00912243	1.253E-010
179	3179.699	IR	0.00280711	9.664E-011	179	3172.046	RA	5.98174e-10	1.701E-010	179	3172.421	IR	0.00253928	2.275E-010
180	3179.717	RA	0.000103368	2.087E-010	180	3172.072	IR	0.0103645	8.885E-011	180	3172.431	RA	2.42535e-07	2.532E-010

Comparison of experimental and simulation data plots

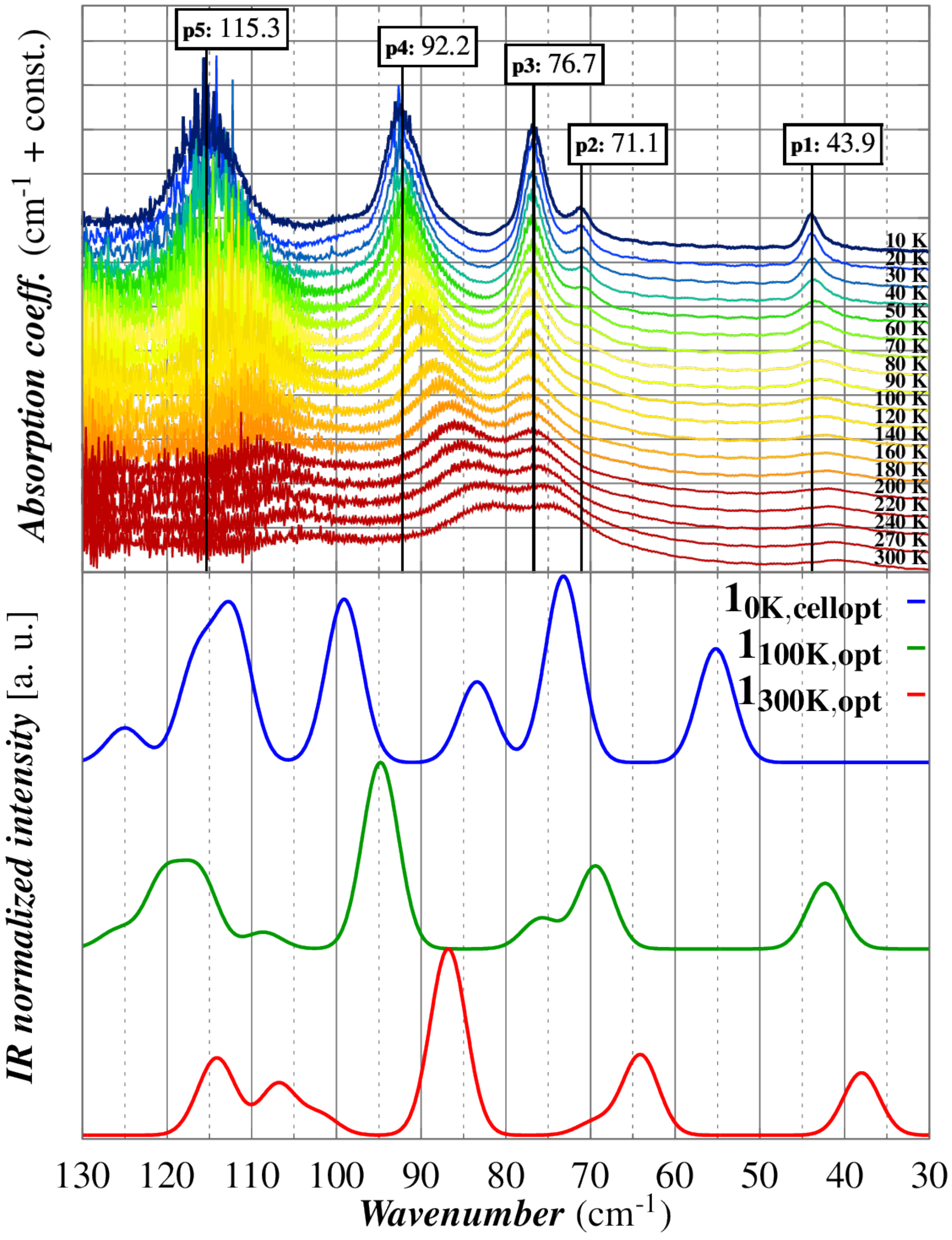

FIG. S 5 Top panel: THz spectra of (**2**) as a function of temperature. Bottom panel: DFT vibrational analysis of $\mathbf{1}_{\mathbf{0K,cellopt}}$ $\mathbf{1}_{\mathbf{100K,opt}}$ and $\mathbf{1}_{\mathbf{300K,opt}}$.

FIG. S 6 Normal modes composition in IR peaks 1 and 2

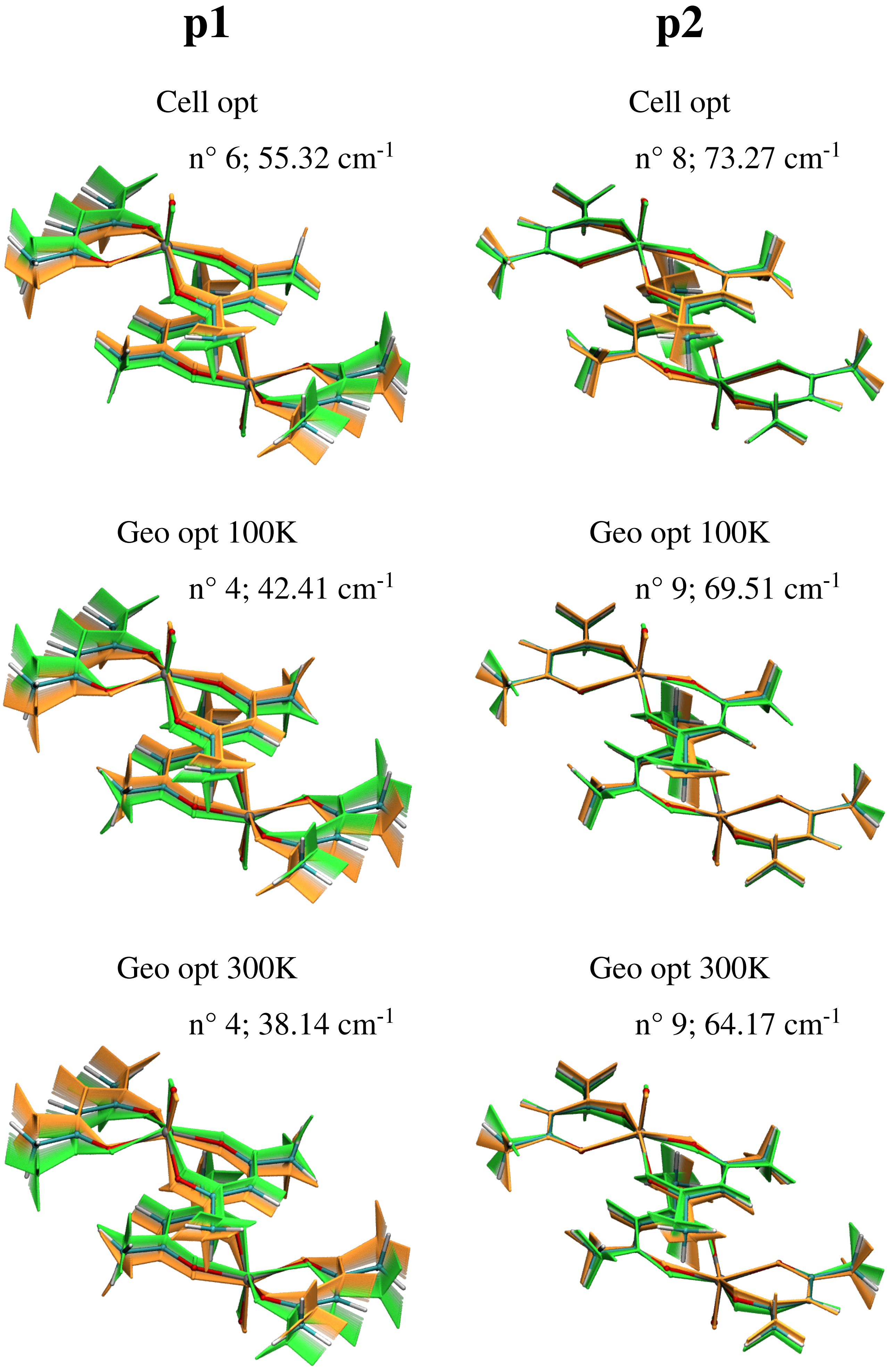

FIG. S 7 Normal modes composition in IR peaks 3 and 4

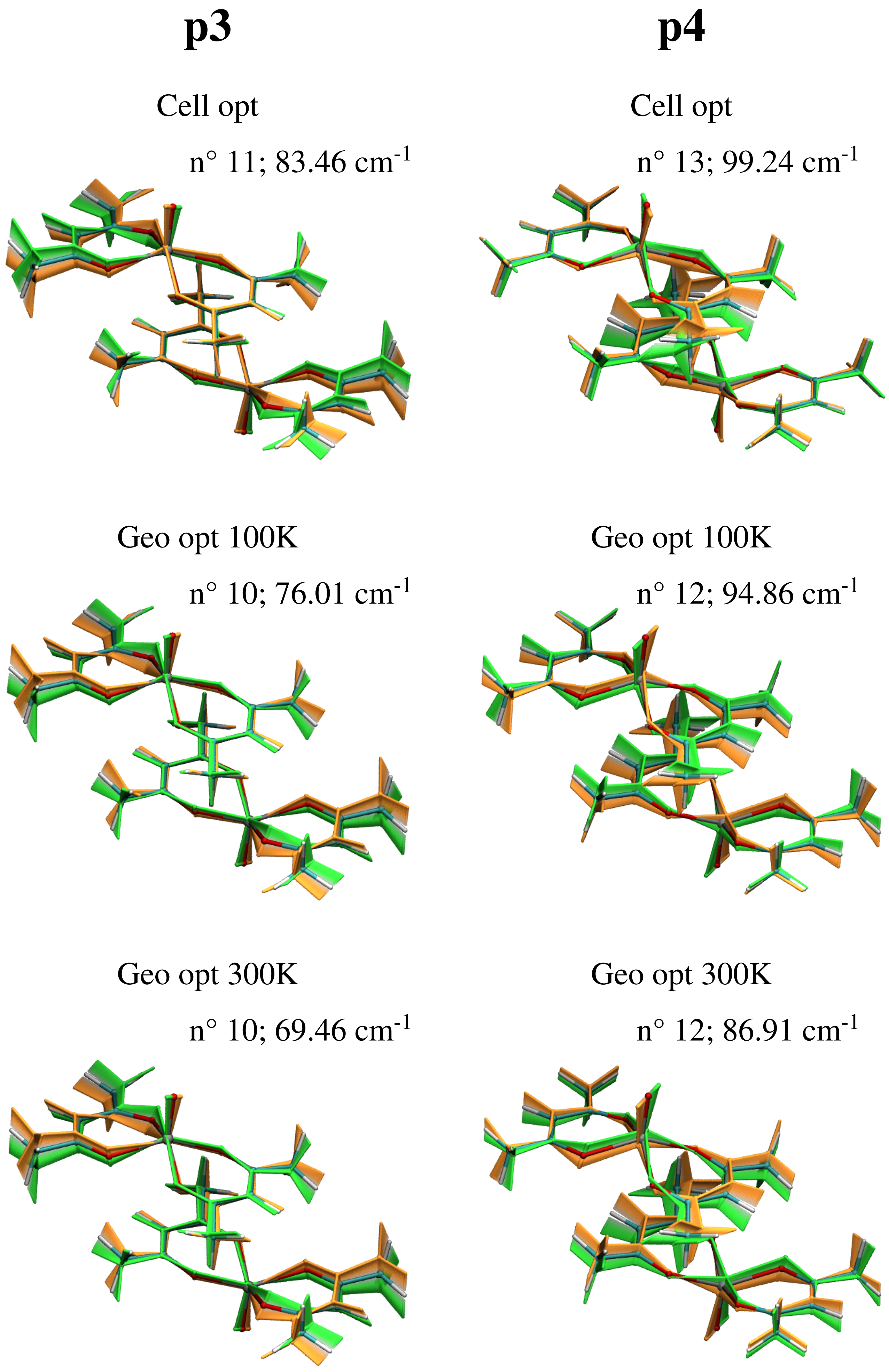

FIG. S 8 Normal modes composition in IR peaks 5

p5

Cell opt

n° 15; 112.35 cm^{-1}

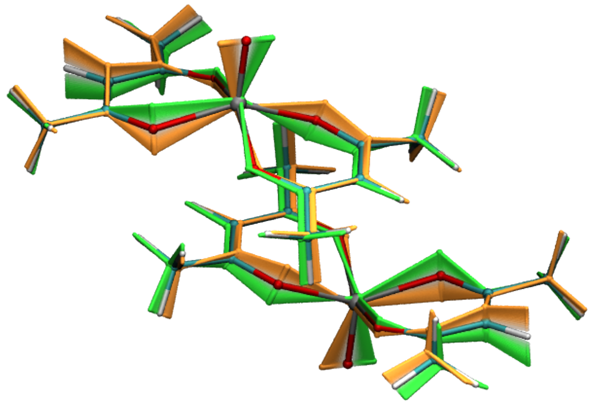

Cell opt

n° 17; 116.56 cm^{-1}

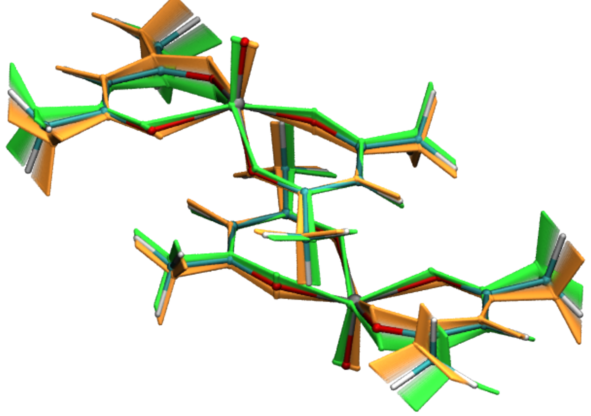

Geo opt 100K

n° 15; 108.69 cm^{-1}

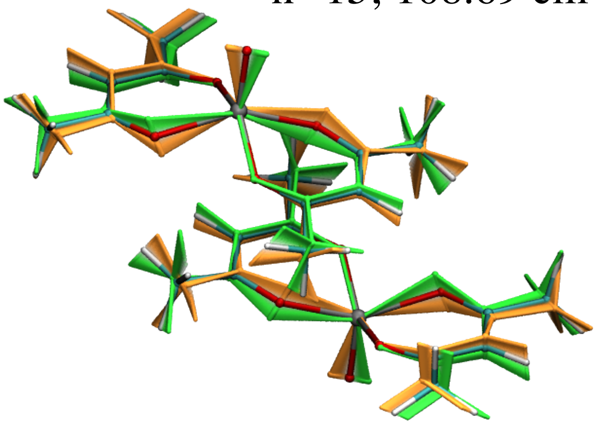

Geo opt 300K

n° 15; 102.07 cm^{-1}

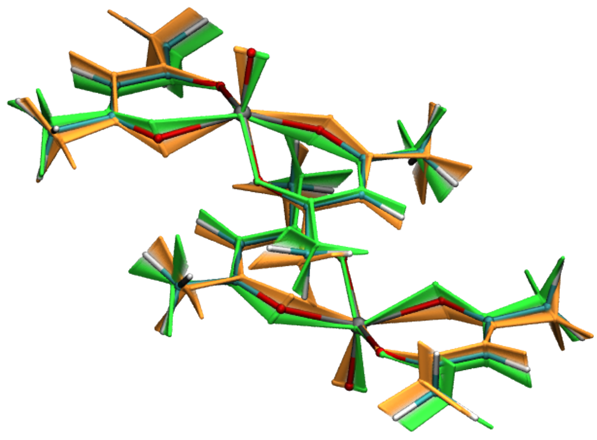

Geo opt 300K

n° 17; 106.96 cm^{-1}

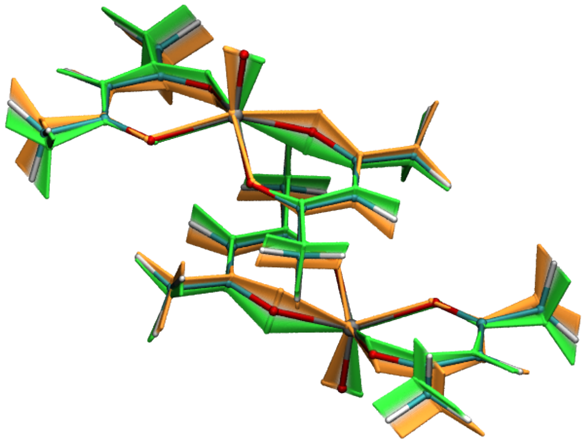

FIG. S 9 Normal modes composition in IR peaks 6

p6

Cell opt

n° 18; 125.11 cm^{-1}

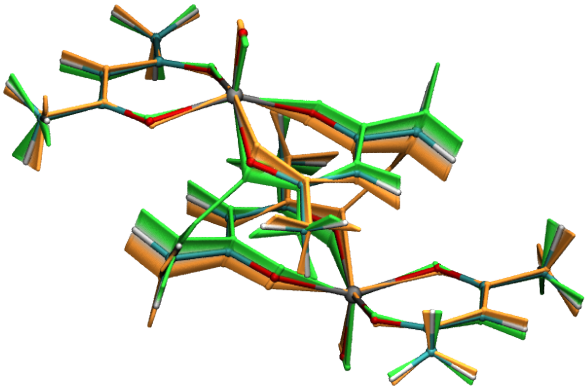

Geo opt 100K

n° 17; 116.26 cm^{-1}

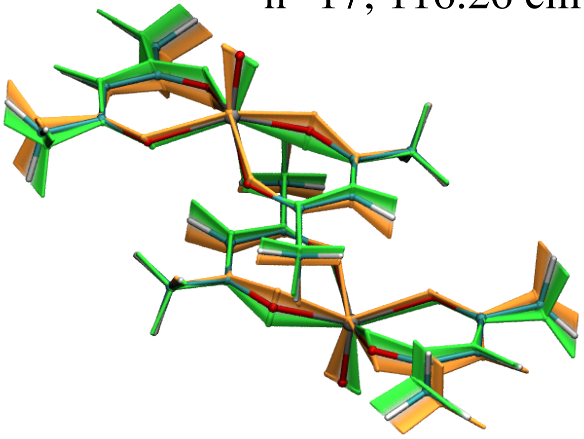

Geo opt 100K

n° 18; 120.49 cm^{-1}

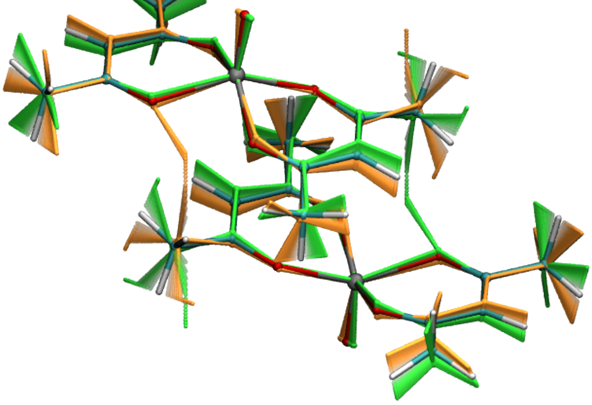

Geo opt 300K

n° 18; 113.79 cm^{-1}

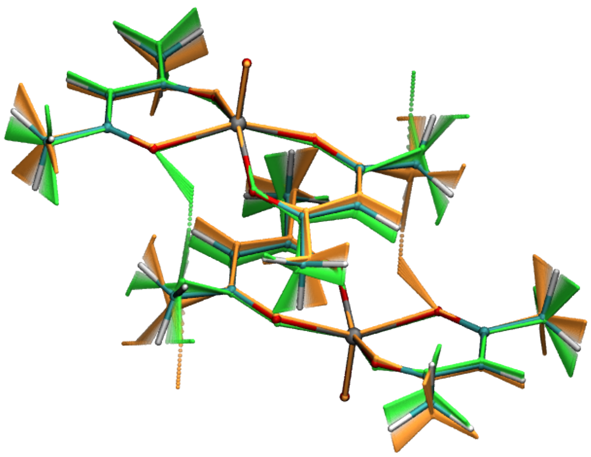

Geo opt 300K

n° 19; 115.08 cm^{-1}

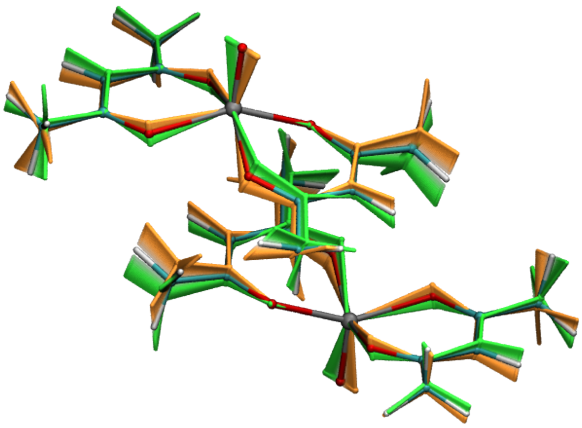

TAB. S 5 Charge variation of one molecule of $[VO(acac)_2]$ crystal is analysed along several normal modes from $\mathbf{1_{0K,cellopt}}$ structure. The metal center and first coordination sphere atomic charges are reported. α is the normal mode index, ω_α is the frequency, q_α represents the progress along the normal coordinate.

α	ω_α	Simm.	Sp-Ph	q_α	V	$O_{vanadyl}$	O_{acac}	O_{acac}	O_{acac}	O_{acac}
–	–	–	–	0.0	0.7052	-0.3686	-0.3011	-0.2890	-0.2829	-0.2932
6	55.32	IR - **p1**	$2.33 \cdot 10^{-7}$	1.0	0.7053	-0.3691	-0.3013	-0.2886	-0.2832	-0.2925
8	73.27	IR - **p2**	$2.33 \cdot 10^{-7}$	1.0	0.7054	-0.3678	-0.3003	-0.2904	-0.2834	-0.2939
9	75.29	Raman	$2.36 \cdot 10^{-7}$	1.0	0.7067	-0.3689	-0.3012	-0.2899	-0.2829	-0.2920
11	83.46	IR - **p3**	$2.45 \cdot 10^{-7}$	1.0	0.7038	-0.3688	-0.3022	-0.2888	-0.2832	-0.2924
45	266.12	IR	$1.81 \cdot 10^{-6}$	1.0	0.7096	-0.3718	-0.3004	-0.2909	-0.2815	-0.2960
62	483.88	Raman	$3.68 \cdot 10^{-6}$	1.0	0.7022	-0.3679	-0.3050	-0.2938	-0.2785	-0.2882
93	1000.96	IR	$8.66 \cdot 10^{-6}$	1.0	0.6949	-0.3438	-0.3021	-0.2907	-0.2839	-0.2958

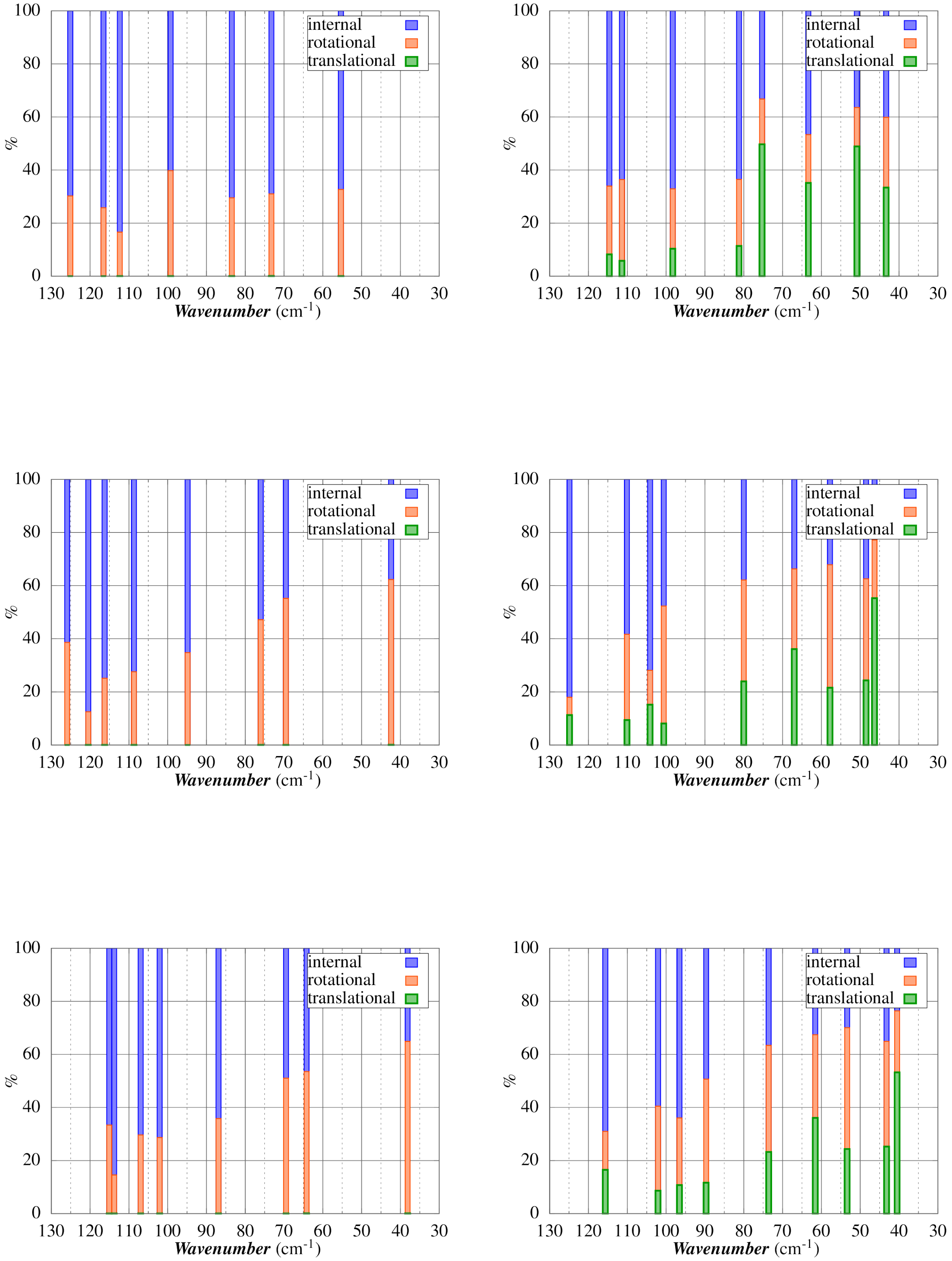

FIG. S 10 Total motion of a single molecule associated with IR (left) and Raman (right) normal modes, from the top $\mathbf{1}_{\mathbf{0K},\mathbf{cellopt}}$, $\mathbf{1}_{\mathbf{100K},\mathbf{opt}}$ and $\mathbf{1}_{\mathbf{300K},\mathbf{opt}}$sets. The modes were decomposed in intramolecular percentage (blue), rotational percentage (orange), and translational percentage (green).

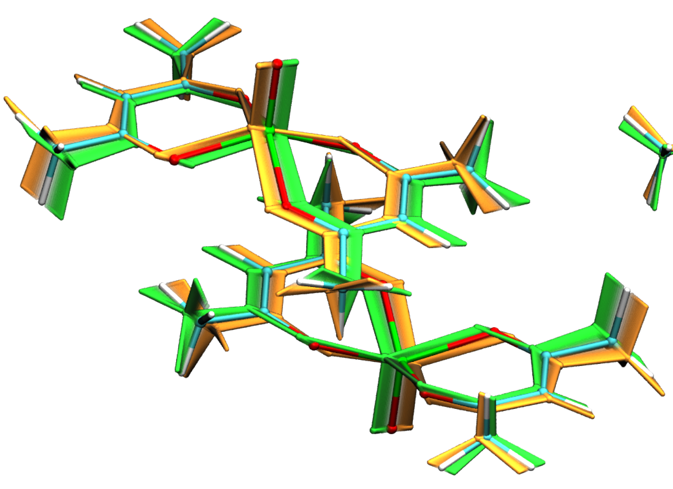

Mode n° 8

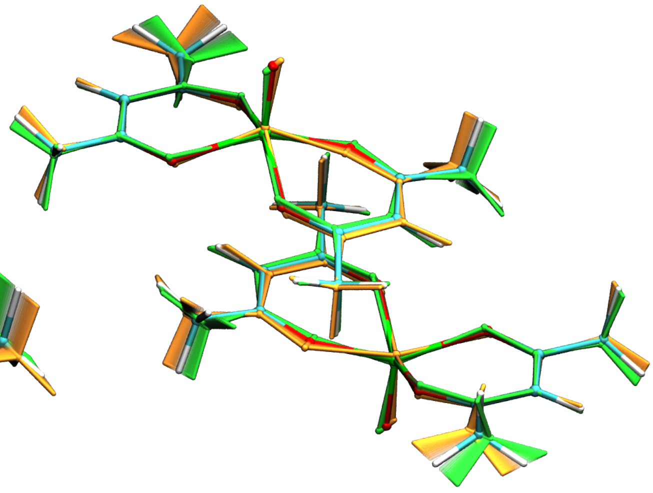

Mode n° 45

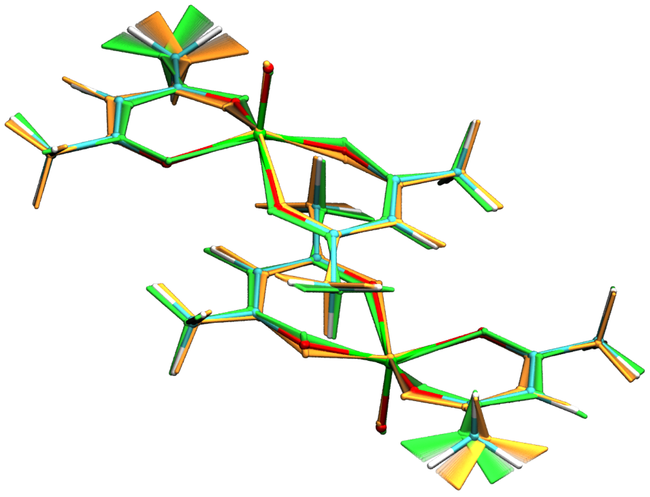

Mode n° 47

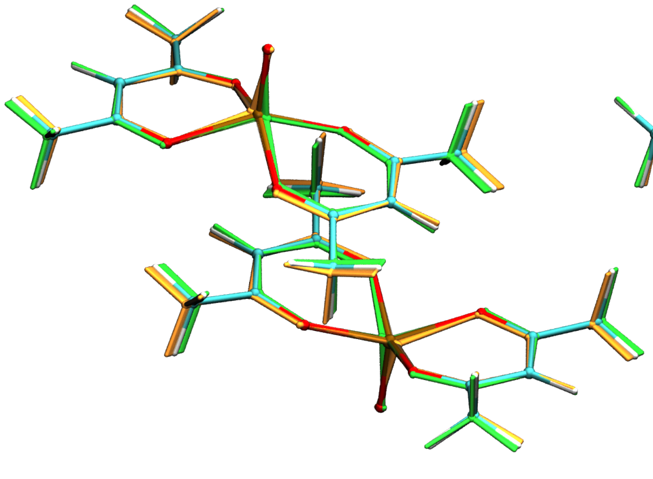

Mode n° 62

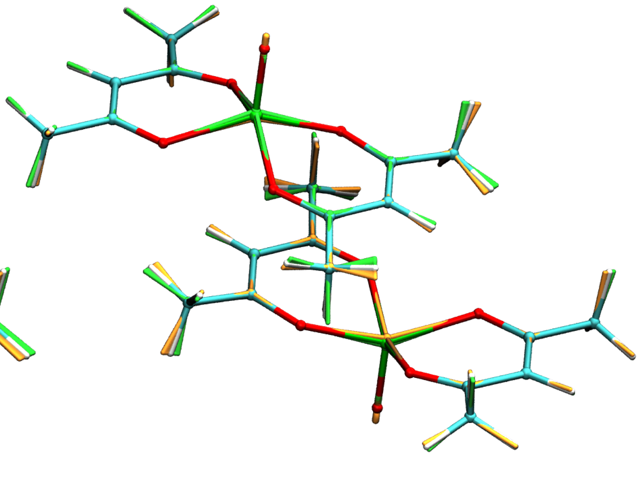

Mode n° 91

FIG. S 11 Normal modes 8, 45, 62, and 91 from $\mathbf{1}_{\mathbf{100K,opt}}$ structure are depicted. 45, 62, and 91 show the largest charges variation and the largest Sp-Ph coupling.

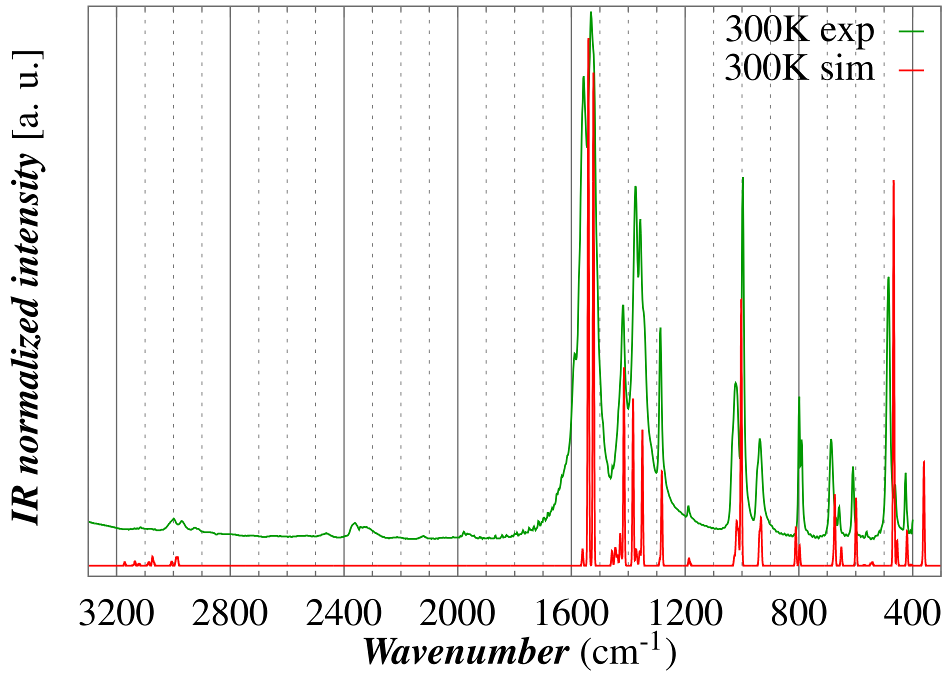

FIG. S 12 400-4000 cm-1 IR measured spectrum (green), simulated 300K spectrum (red)
